# Errors in Statistical Inference Under Model Misspecification: Evidence, Hypothesis Testing, and AIC

**DOI:** 10.3389/fevo.2019.00372

**Published:** 2019-10-21

**Authors:** Brian Dennis, José Miguel Ponciano, Mark L. Taper, Subhash R. Lele

**Affiliations:** 1 Department of Fish and Wildlife Sciences and Department of Statistical Science, University of Idaho, Moscow, ID, United States,; 2 Biology Department, University of Florida, Gainesville, FL, United States,; 3 Department of Ecology, Montana State University, Bozeman, MT, United States,; 4 Department of Mathematical and Statistical Sciences, University of Alberta, Edmonton, AB, Canada

**Keywords:** model misspecification, evidential statistics, evidence, error rates in model selection, Kullback-Leibler divergence, hypothesis testing, Akaike’s information criterion, model selection

## Abstract

The methods for making statistical inferences in scientific analysis have diversified even within the frequentist branch of statistics, but comparison has been elusive. We approximate analytically and numerically the performance of Neyman-Pearson hypothesis testing, Fisher significance testing, information criteria, and evidential statistics (Royall, 1997). This last approach is implemented in the form of evidence functions: statistics for comparing two models by estimating, based on data, their relative distance to the generating process (i.e., truth) (Lele, 2004). A consequence of this definition is the salient property that the probabilities of misleading or weak evidence, error probabilities analogous to Type 1 and Type 2 errors in hypothesis testing, all approach 0 as sample size increases. Our comparison of these approaches focuses primarily on the frequency with which errors are made, both when models are correctly specified, and when they are misspecified, but also considers ease of interpretation. The error rates in evidential analysis all decrease to 0 as sample size increases even under model misspecification. Neyman-Pearson testing on the other hand, exhibits great difficulties under misspecification. The real Type 1 and Type 2 error rates can be less, equal to, or greater than the nominal rates depending on the nature of model misspecification. Under some reasonable circumstances, the probability of Type 1 error is an increasing function of sample size that can even approach 1! In contrast, under model misspecification an evidential analysis retains the desirable properties of always having a greater probability of selecting the best model over an inferior one and of having the probability of selecting the best model increase monotonically with sample size. We show that the evidence function concept fulfills the seeming objectives of model selection in ecology, both in a statistical as well as scientific sense, and that evidence functions are intuitive and easily grasped. We find that consistent information criteria are evidence functions but the MSE minimizing (or efficient) information criteria (e.g., AIC, AICc, TIC) are not. The error properties of the MSE minimizing criteria switch between those of evidence functions and those of Neyman-Pearson tests depending on models being compared.

## 1. INTRODUCTION

### 1.1. Background

In the twentieth century, the bulk of scientific statistical inference was conducted with Neyman-Pearson hypothesis tests, a term which we broadly take to encompass significance testing, *P*-values, generalized likelihood ratio, and other special cases, adaptations, or generalizations. The central difficulty with interpreting NP tests is that the Type 1 error probability (usually denoted *α*) remains fixed regardless of sample size, rendering problematic the question of what constitutes evidence *for* the model serving as the null hypothesis (Aho et al., 2014; Murtaugh, 2014; Spanos, 2014). The fixed null error rate of hypothesis testing lies at the core of why model selection procedures based on hypothesis testing (such as stepwise regression and multiple comparisons) have always had the reputation of being jury-rigged contraptions that have never been fully satisfactory (Gelman et al., 2012). An additional problem with hypothesis tests arises from the “Type 3” error of model misspecification, in which neither the null nor the alternative hypothesis model adequately describes the data (Mosteller, 1948). The influence of model misspecification on all types of inference is under appreciated.

A substantial advance in late 20th century statistical practice was the development of information-theoretic indexes for model selection, namely the Akaike information criterion (AIC) and its variants (Akaike, 1973, 1974; Sakamoto et al., 1986; Bozdogan, 1987). The model selection criteria were slow in coming to ecology (Kemp and Dennis, 1991; Lebreton et al., 1992; Anderson et al., 1994; Strong et al., 1999) but have rapidly proliferated in the past 20 years, aided by a popular book (Burnham and Anderson, 2002) and journal reviews (Anderson et al., 2000; Johnson and Omland, 2004; Ward, 2008; Grueber et al., 2011; Symonds and Moussalli, 2011). Ecological practice has been indelibly shaped by the use of AIC and similar indexes (Guthery et al., 2005; Barker and Link, 2015). Notwithstanding, ecologists, traditionally introspective about and scrutinizing of statistical practices (Strong, 1980; Quinn and Dunham, 1983; Loehle, 1987; Yoccoz, 1991; Johnson, 1999; Hurlbert and Lombardi, 2009; Gerrodette, 2011), have generated much critique and discussion of the appropriate uses of the information criteria (Guthery et al., 2005; Richards, 2005; Arnold, 2010; Barker and Link, 2015; Cade, 2015). Topics of discussions have focused on the contrast of information-theoretic methods with frequentist hypothesis testing methods (Anderson et al., 2000; Stephens et al., 2005; Murtaugh, 2009) and with Bayesian statistical approaches (Link and Barker, 2006; Barker and Link, 2015).

In an apparently separate statistical development, the concept of statistical evidence was refined in light of the shortcomings of using as evidence quantities such as *P*-values that emerge from frequentist hypothesis testing (Royall, 1997, 2000; Taper and Lele, 2004, 2011; Taper and Ponciano, 2016). Crucial to the evidence concept was the idea of an evidence function (Lele, 2004). An evidence function is a statistic for comparing two models that has a suite of statistical properties, among them two critical properties: (a) both error probabilities (analogous to Type 1 and Type 2 error probabilities in hypothesis testing) approach zero asymptotically as the sample size increases, and (b) when the models are misspecified and the concept of “error” is generalized to be the selection of the model “farthest” from the true data-generating process, the two error probabilities still approach zero as sample size increases.

Despite widespread current usage of AIC-type indexes in ecology, the inferential basis and implications of the use of information criteria are not fully developed, and what is developed is commonly misunderstood (see the forum edited by Ellison et al., 2014). AIC-type indexes are used for different purposes: in some contexts in place of hypothesis testing, in some as evidence for model identification, in some as estimates of pseudo-Bayesian model probabilities, and in some purely as criteria for prediction (Anderson et al., 2001). Of concern is that few ecologists can explain the inferences they are conducting with AIC, as Akaike’s (Akaike, 1973, 1974) mathematical argument is not an easy one, and more recent accounts (Bozdogan, 1987; Burnham and Anderson, 2002; Claeskens and Hjort, 2008) are heavily mathematical as well. A clear and accessible inferential concept is needed to promote confidence in and appropriate uses of the information-theoretic criteria. We believe that the concept of statistical evidence can serve well as the inferential basis for the uses of and distinctions among the AIC-type indexes.

This paper contrasts the concept of evidence with classical statistical hypothesis testing and demonstrates that many information-based indexes for model selection can be recast and interpreted as evidence functions. We show that the evidence function concept fulfills many seeming objectives of model selection in ecology, both in a statistical as well as scientific sense, and that evidence functions are intuitive and easily grasped. Specifically, the difference of two values of an information-theoretic index for a pair of models possesses in whole or in part the properties of an evidence function and thereby grants to the resulting inference a scientific warrant of considerable novelty in ecological practice.

Of particular importance is the desirable behavior of evidence functions under model misspecification, behavior which, as we shall show, departs sharply from that of statistical hypothesis testing. As ecologists grapple increasingly with issues related to multiple quantitative hypotheses for how data arose, the evidence function concept can serve as a scientifically satisfying basis for model comparison in observational and experimental studies.

### 1.2. Method of Analysis and Notation

For convenience we label as Neyman-Pearson (NP) hypothesis tests a broad collection of interrelated statistical inference techniques, including *P*-values for likelihood ratios, confidence intervals, and generalized likelihood ratio tests, that are connected to Neyman and Pearson’s original work (Neyman and Pearson, 1933) and that form the core of modern applied statistics. We distinguish Fisher’s use of *P*-values as a measure of the adequacy of the null hypothesis from the use of *P*-values in likelihood ratio hypothesis tests.

NP hypothesis tests and evidential comparisons are conducted in very different fashions and operate under different warrants. Thus, comparison is difficult. However, they both make inferences. One fundamental metric by which they can be compared is the frequency that inferences are made in error. In this paper we seek to illuminate how the frequency of errors made by these methods is influenced by sample size, the differences among models being compared, and also the differences between candidate models and the true data generating process. Both of these inferential approaches can be, and generally are, constructed around a base of the likelihood ratio (LR). By studying the statistical behavior of the LR, we can answer our questions regarding frequency of error in all approaches considered.

Throughout this discussion, one observation (datum) is represented using the random variable *X* with *g*(*x*) being the probability density function representing the true, data-generating process and *f* (*x*) being the probability density function of an approximating model. If the observed process is discrete, *g*(*x*) and *f* (*x*) will represent probability mass functions. For simplicity we refer to these functions in both the discrete and continuous cases as pdf’s, thinking of the abbreviation as “probability distribution function.” The likelihood function under the true model, for *n* independent and identically distributed (*iid*) observations *x*_1_, *x*_2_, … *x*_*n*_ is written as

(1) $$L_{g}=g\left(x_{1}\right)g\left(x_{2}\right)\ldots g\left(x_{n}\right),$$

whereas under the approximating model it is

(2) $$L_{f}=f\left(x_{1}\right)f\left(x_{2}\right)\ldots f\left(x_{n}\right).$$

In cases where there are two candidate models *f*_1_(*x*) and *f*_2_(*x*), we write the respective likelihoods as *L*_1_ and *L*_2_ to avoid double subscript levels.

We make much use of the Kullback-Leibler (KL) divergence, one of the most commonly used measures of the difference between two distributions. The KL divergence of *f* (*x*) from *g*(*x*), denoted *K*(*g*, *f*), is defined as the expected value of the log-likelihood ratio of *g* and *f* (for one observation) given that the observation came from the process represented by *g*(*x*):

(3) $$K(g,f)\equiv {\text{E}}_{g}\left[\text{log}\left(\frac{g(X)}{f(X)}\right)\right]=\int \sum g(x)\text{log}\left(\frac{g(x)}{f(x)}\right).$$

Here E_*g*_ denotes expectation with respect to the distribution represented by *g*(*x*). The expectation is a sum or integral (or both) over the entire range of the random variable *X*, depending on whether the probability distributions represented by *g*(*x*) and *f* (*x*) are discrete or continuous (or both, such as for a zero-inflated continuous distribution). The functions must give positive probability to the same sets (along with other technical mathematical requirements which are usually met by the common models of ecological statistics).

The KL divergence is interpreted as the amount of information lost when using model *f* (*x*) to approximate the data generating process *g*(*x*) (Burnham and Anderson, 2001). Its publication (Kullback and Leibler, 1951) was a highpoint in the golden age of the study of “information theory.” The KL divergence is always positive if *g*(*x*) and *f* (*x*) represent different distributions and is zero if the distributions are identical (“identical” in the mathematical sense that the distributions give the same probabilities for all events in the sample space). The KL divergence is not a mathematical distance measure in that *K*(*g*, *f*) is not in general equal to *K*(*f*, *g*).

The relevant KL divergences under correct model specification are for *f*_1_(*x*) and *f*_2_(*x*) with respect to each other:

(4) $$K_{12}\equiv K\left(f_{1},f_{2}\right)={\text{E}}_{1}\left[\text{log}\left(\frac{f_{1}(X)}{f_{2}(X)}\right)\right]=\int \sum f_{1}(x)\text{log}\left(\frac{f_{1}(x)}{f_{2}(x)}\right),$$

(5) $$K_{21}\equiv K\left(f_{2},f_{1}\right)={\text{E}}_{2}\left[\text{log}\left(\frac{f_{2}(X)}{f_{1}(X)}\right)\right]=\int \sum f_{2}(x)\text{log}\left(\frac{f_{2}(x)}{f_{1}(x)}\right).$$

By reversing numerator and denominator in the log function in Equation (5), one finds that

(6) $${\text{E}}_{2}\left[\text{log}\left(\frac{f_{1}(X)}{f_{2}(X)}\right)\right]=\int \sum f_{2}(x)\text{log}\left(\frac{f_{1}(x)}{f_{2}(x)}\right)=-K_{21}.$$

The convention for which subscript is placed first varies among references; we put the subscript of the reference distribution first as it is easy to remember.

The likelihood ratio (LR) and its logarithm figure prominently in statistical hypothesis testing as well as in evidential statistics. The LR is

(7) $$\frac{L_{1}}{L_{2}}=\frac{f_{1}\left(x_{1}\right)f_{1}\left(x_{2}\right)\cdots f_{1}\left(x_{n}\right)}{f_{2}\left(x_{1}\right)f_{2}\left(x_{2}\right)\cdots f_{2}\left(x_{n}\right)},$$

and the log-LR is

(8) $$\text{log}\left(\frac{L_{1}}{L_{2}}\right)=\overset{n}{\underset{i=1}{\sum }}\text{log}\left(\frac{f_{1}\left(x_{i}\right)}{f_{2}\left(x_{i}\right)}\right).$$

In particular, the log-LR considered as a random variable is a sum of iid random variables, and its essential statistical properties can be approximated using the central limit theorem (CLT). The CLT (Box 1) provides an approximate normal distribution for a sum of iid random variables and requires the expected value (mean) and the variance of one of the variables. Under correct model specification, the observations came from either *f*_1_(*x*) or *f*_2_(*x*), and Equations (4)–(6) above give the expected value of one of the random variables in the sum as *K*_12_ or −*K*_21_, depending on which model generated the data. Let $\sigma_{1}^{2}$ and $\sigma_{2}^{2}$ be the variances of log [*f*_1_ (*X*)/*f*_2_ (*X*)] with respect to each model:

(9) $$\sigma_{1}^{2}={\text{V}}_{1}\left[\text{log}\left(\frac{f_{1}(X)}{f_{2}(X)}\right)\right]=\int \sum f_{1}(x){\left(\text{log}\left(\frac{f_{1}(x)}{f_{2}(x)}\right)\right)}^{2}-{K_{12}}^{2}.$$

(10) $$\sigma_{2}^{2}={\text{V}}_{2}\left[\text{log}\left(\frac{f_{1}(X)}{f_{2}(X)}\right)\right]=\int \sum f_{2}(x){\left(\text{log}\left(\frac{f_{1}(x)}{f_{2}(x)}\right)\right)}^{2}-{K_{21}}^{2}.$$

One can envision cases in which these variances might not exist, but we do not consider such cases here. The CLT, which requires that the variances be finite, provides the following approximations. If the data arise from *f*_1_:

(11) $$\text{log}\left(\frac{L_{1}}{L_{2}}\right)\dot{\sim }\text{normal}\left(nK_{12},n{\sigma_{1}}^{2}\right),$$

(12) $$\frac{1}{n}\text{log}\left(\frac{L_{1}}{L_{2}}\right)\dot{\sim }\text{normal}\left(K_{12},{\sigma_{1}}^{2}/n\right),$$

(13) $$\frac{\sqrt{n}}{\sigma_{1}}\left[\frac{1}{n}\text{log}\left(\frac{L_{1}}{L_{2}}\right)-K_{12}\right]\dot{\sim }\text{normal}(0,1).$$

Here, ⩪ means “is approximately distributed as.” If the data arise from *f*_2_:

(14) $$\text{log}\left(\frac{L_{1}}{L_{2}}\right)\dot{\sim }\text{normal}\left(-nK_{21},n{\sigma_{2}}^{2}\right),$$

(15) $$\frac{1}{n}\text{log}\left(\frac{L_{1}}{L_{2}}\right)\dot{\sim }\text{normal}\left(-K_{21},{\sigma_{2}}^{2}/n\right),$$

(16) $$\frac{\sqrt{n}}{\sigma_{2}}\left[\frac{1}{n}\text{log}\left(\frac{L_{1}}{L_{2}}\right)+K_{21}\right]\dot{\sim }\text{normal}(0,1).$$

The device of using the CLT to study properties of the likelihood ratio is old and venerable and figures prominently in the theory of sequential statistical analysis (Wald, 1945).

A model, *f*, can be said to be misspecified if the distribution of data implied by the model (under best possible parameterization) differs in any way from the distribution of data under the true generating process. In the Kullback-Leibler divergence setting within which we are working, *f* is misspecified if *K*(*g*, *f*) > 0. A model set can be said to be misspecified if all of its member models are misspecified. Misspecification can have a host of causes, including omission of real covariates, inclusion of spurious covariates, incorrect specification of functional form, incorrect specification of process error structure, and incorrect specification of measurement error structure.

The approximate behavior of the LR under misspecification can also be represented with the CLT. To our two model candidates *f*_1_ (*x*) and *f*_2_ (*x*), we add the pdf *g* (*x*) defined as the best possible mathematical representation of the distribution of data stemming from the actual stochastic mechanism generating the data, the unknown “truth” sought by scientists. We denote by Δ*K* the difference of the KL divergences of *f*_1_(*x*) or *f*_2_ (*x*), from *g* (*x*):

(17) $$\Delta K=K\left(g,f_{2}\right)-K\left(g,f_{1}\right).$$

We note that Δ*K* could be positive, negative, or zero: if Δ*K* is positive, then *f*_1_ is “closer” to truth, if Δ*K* is negative, then *f*_2_ is closer to truth, and if Δ*K* is zero, then both models are equally distant from truth. To deploy the CLT, we need the mean and variance of the single-observation LR under misspecification. For the mean we have

(18) $${\text{E}}_{g}\left[\text{log}\left(\frac{f_{1}(X)}{f_{2}(X)}\right)\right]=\int \sum g(x)\text{log}\left(\frac{f_{1}(x)}{f_{2}(x)}\right)=\Delta K$$

The rightmost equality is established by adding and subtracting E*g* [log (*g* (*X*)]. We denote the variance by *σ*_*g*_^2^ which becomes

(19) $${\text{V}}_{g}\left[\text{log}\left(\frac{f_{1}(X)}{f_{2}(X)}\right)\right]\equiv {\sigma_{g}}^{2}=\int \sum g(x){\left(\text{log}\left(\frac{f_{1}(x)}{f_{2}(x)}\right)\right)}^{2}-{\left(\Delta K\right)}^{2}.$$

And now by the CLT, if the data did not arise from *f*_1_ (*x*) or *f*_2_ (*x*), but rather from some other pdf *g* (*x*), we have:

(20) $$\text{log}\left(\frac{L_{1}}{L_{2}}\right)\dot{\sim }\text{normal}\left(n\Delta K,n{\sigma_{g}}^{2}\right),$$

(21) $$\frac{1}{n}\text{log}\left(\frac{L_{1}}{L_{2}}\right)\dot{\sim }\text{normal}\left(\Delta K,{\sigma_{g}}^{2}/n\right),$$

(22) $$\frac{\sqrt{n}}{\sigma_{g}}\left[\frac{1}{n}\text{log}\left(\frac{L_{1}}{L_{2}}\right)-\Delta K\right]\dot{\sim }\text{normal}(0,1).$$

**FIGURE 1 |:**
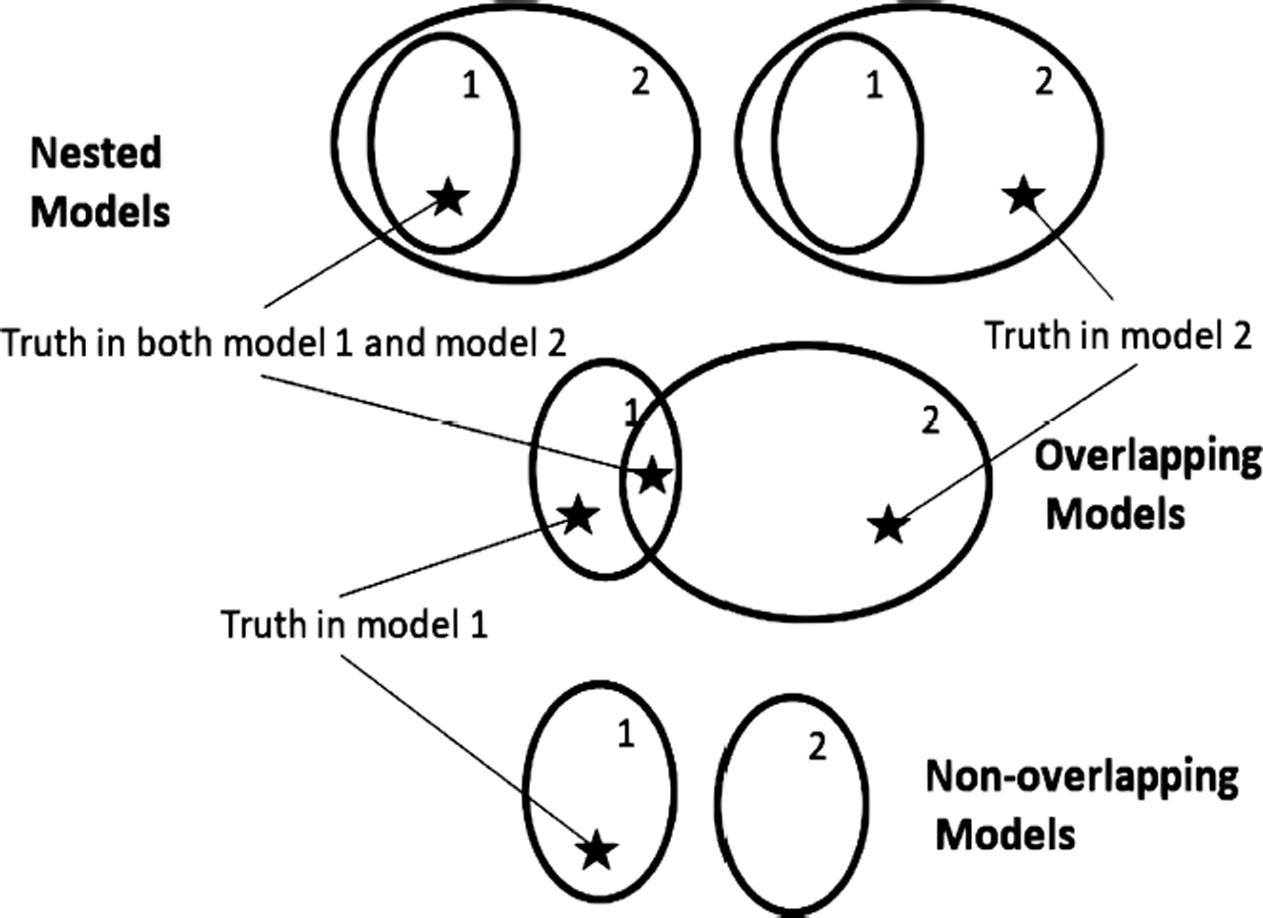
Model topologies when models are correctly specified. Regions represent parameter spaces. Star represents the true parameter value corresponding to the model that generated the data. **Top**: a nested configuration would occur, for example, in the case of two regression models if the first model had predictor variables *R*_1_ and *R*_2_ while the second had predictor variables *R*_1_, *R*_2_, and *R*_3_. **Middle**: an overlapping configuration would occur if the first model had predictor variables *R*_1_ and *R*_2_ while the second had predictor variables *R*_2_ and *R*_3_. Three locations of truth are possible: truth in model 1, truth in model 2, and truth in both models 1 and 2. **Bottom**: an example of a non-overlapping configuration is when the first model has predictor variables *R*_1_ and *R*_2_ while the second model has predictor variables *R*_3_ and *R*_4_.

**FIGURE 2 |:**
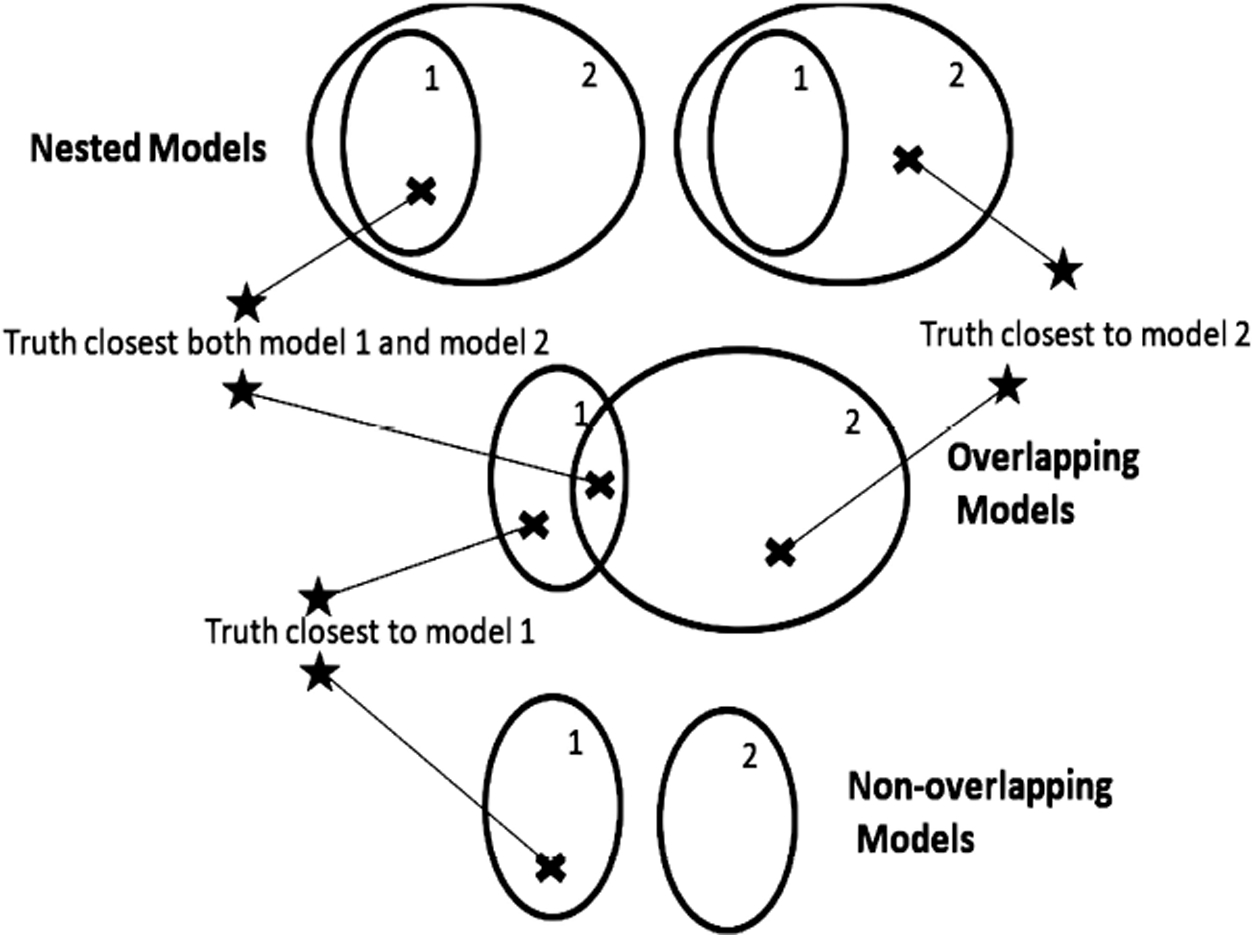
Model topologies when models are misspecified. Regions represent parameter spaces. Star represents the true model that generated the data. Exes represent the point in the parameter space covered by the model set closest to the true generating process.

Critical to the understanding, both mathematical and intuitive, of inference on models is an understanding of the topology of models. Once one has a concept of distances between models, a topology is implied. A model with one or more unknown parameters represents a whole family or set of models, with each parameter value giving a completely specified model. At times we might refer to a model set as a model if there is no risk of confusion. Two model sets can be only be arranged as nested, overlapping, or non-overlapping. A set of models can be correctly specified or misspecified depending on whether or not the generating process can be exactly represented by a model in the model set. Thus, there are only six topologies relating two model sets to the generating process (Figures 1, 2).

## 2. EVIDENCE, NEYMAN-PEARSON TESTING, AND FISHER SIGNIFICANCE

### 2.1. Correctly Specified Models

In the canon of traditional statistical practices for comparing two candidate models, *f*_1_ (*x*) and *f*_2_ (*x*) say, with or without unknown parameters involved, the assumption that the data arose from either *f*_1_ (*x*) or *f*_2_ (*x*) is paramount. In this section we adopt this assumption of correctly specified models and compare the properties of statistical hypothesis testing with those of the evidence approach. The correct model assumption is the home turf, so to speak, of hypothesis testing, and so the comparison should by rights highlight the strengths of traditional statistical practice. To focus the issues with clarity we concentrate on the case in which *f*_1_ (*x*) and *f*_2_ (*x*) are statistically simple hypotheses (a.k.a. completely specified models, not to be confused with correctly specified models). In other words, we assume for now there are no unknown parameters in either model, deferring until later in this paper a discussion of unknown parameters.

#### 2.1.1. Neyman-Pearson Statistical Hypothesis Tests

Neyman and Pearson (1933) proved in a famous theorem (the “Neyman-Pearson Lemma”) that basing a decision between two completely specified hypotheses (H_1_: the data arise from model *f*_1_ (*x*), and H_2_: the data arise from model *f*_2_ (*x*)) on the likelihood ratio had certain optimal properties. Neyman and Pearson’s LR decision rule has the following structure:

(23) $$\text{decide}\;\text{on}\;{\text{H}}_{1}\;\text{if}\;L_{1}/L_{2}>c,\;\text{decide}\;\text{on}\;{\text{H}}_{2}\;\text{if}\;L_{1}/L_{2}>c.$$

Here the cutoff quantity (or critical value) *c* is determined by setting an error probability equal to a known constant (usually small), denoted *α*. Specifically, the conditional probability of wrongly deciding on H_2_ given that H_1_ is true is the “Type 1 error probability” and is denoted as *α*.

(24) $$\text{P}\left(L_{1}/L_{2}\leq c|{\text{H}}_{1}\right)=\alpha .$$

Often for notational convenience in lieu of the statement “H_*i*_ is true” we will simply write “H_*i*_.” Now, such a data-driven decision with fixed Type 1 error probability is the traditional form of a statistical hypothesis test. A test with a Type 1 error probability of *α* is said to be a size *α* test. The other error probability (“Type 2”), the conditional probability of wrongly deciding on H_1_ given H_2_, is usually denoted *β*:

(25) $$\text{P}\left(L_{1}/L_{2}>c|{\text{H}}_{2}\right)=\beta$$

The power of the test is defined as the quantity 1 − *β*. Neyman and Pearson’s theorem, stating that no other test of size *α* or less has power that can exceed the power of the likelihood ratio test, is a cornerstone of most contemporary introductions to mathematical statistics (Rice, 2007; Samaniego, 2014).

With the central limit theorem results (Equations 11–16), the error properties of the NP test can be approximated. To find the critical value *c*, we have under H_1_:

(26) $$\frac{L_{1}}{L_{2}}\leq c\Rightarrow \frac{\sqrt{n}}{\sigma_{1}}\left[\frac{1}{n}\text{log}\left(\frac{L_{1}}{L_{2}}\right)-K_{12}\right]\leq \frac{\sqrt{n}}{\sigma_{1}}\left[\frac{1}{n}\text{log}(c)-K_{12}\right],$$

and so the CLT tells us that

(27) $$\alpha =\text{P}\left(\frac{L_{1}}{L_{2}}\leq c|{\text{H}}_{1}\right)\approx \Phi \left(\frac{\sqrt{n}}{\sigma_{1}}\left[\frac{1}{n}\text{log}(c)-K_{12}\right]\right),$$

where Φ (*z*) is the cumulative distribution function (cdf) of the standard normal distribution. The approximate critical value *c* required for a size *α* test is then found by solving Equation (27) for *c*:

(28) $$\Phi \left(\frac{\sqrt{n}}{\sigma_{1}}\left[\frac{1}{n}\text{log}(c)-K_{12}\right]\right)=\alpha \;\Rightarrow \frac{\sqrt{n}}{\sigma_{1}}\left[\frac{1}{n}\text{log}(c)-K_{12}\right]=\Phi^{-1}(\alpha )=-z_{\alpha }\;\Rightarrow c=\text{exp}\left[\sqrt{n}\left(\sqrt{n}K_{12}-\sigma_{1}z_{\alpha }\right)\right].$$

Here *z*_*α*_ = Φ^−1^ (1 − *α*) = −Φ^−1^ (*α*) is the value of the 1 − *α* quantile of the standard normal distribution. Thus, for error rate *α* to be fixed, the critical value as a function of *n* is seen to be a rapidly moving target.

The error probability *β* is approximated in similar fashion. We have, under H_2_,

(29) $$\frac{L_{1}}{L_{2}}>c\Rightarrow \frac{\sqrt{n}}{\sigma_{2}}\left[\frac{1}{n}\text{log}\left(\frac{L_{1}}{L_{2}}\right)+K_{21}\right]>\frac{\sqrt{n}}{\sigma_{2}}\left[\frac{1}{n}\text{log}(c)+K_{21}\right]\;\Rightarrow \frac{\sqrt{n}}{\sigma_{2}}\left[\frac{1}{n}\text{log}\left(\frac{L_{1}}{L_{2}}\right)+K_{21}\right]>\frac{\sqrt{n}}{\sigma_{2}}\left[\frac{1}{n}\text{log}(c)+K_{21}\right],$$

so that, after substituting for *c*,

(30) $$\beta =\text{P}\left(\frac{L_{1}}{L_{2}}>c|{\text{H}}_{2}\right)\approx 1-\Phi \left(\frac{\sqrt{n}}{\sigma_{2}}\left(K_{12}+K_{21}\right)-\frac{\sigma_{1}}{\sigma_{2}}z_{\alpha }\right)\;=\Phi \left(\frac{\sigma_{1}}{\sigma_{2}}z_{\alpha }-\frac{\sqrt{n}}{\sigma_{2}}\left(K_{12}+K_{21}\right)\right).$$

It is seen that *β* → 0 as sample size *n* becomes large. Here *K*_12_ + *K*_21_ is an actual distance measure between *f*_1_ (*x*) and *f*_2_ (*x*) (Kullback and Leibler (1951); sometimes referred to as the “symmetric” KL distance) and can be regarded as the “effect size” as used in statistical power calculations.

Five important points about the Neyman-Pearson Lemma are pertinent here. First, the theorem itself is just a mathematical result and leaves unclear how it is to be used in scientific applications. The prevailing interpretation that emerged in the course of 20^*th*^ century science was that one of the hypotheses, H_1_, would be accorded a special status (“the null hypothesis”), having its error probability *α* fixed at a known (usually small) value by the investigator. The other hypothesis, H_2_, would be set up by experiment or survey design to be the only reasonable alternative to H_1_. The other error probability, *β*, would be managed by study design characteristics (especially sample size), but would remain unknown and could at best only be estimated when the model contained parameters with unknown values. The hypothesis H_1_ would typically play the role of the skeptic’s hypothesis, as in the absence of an effect (absence of a difference in means, absence of influence of a predictor variable, absence of dependence of two categorical variables, etc.) under study. The other hypothesis, H_2_, contains the effect under study and serves as the hypothesis of the researcher, who has the scientific charge of convincing a reasoned skeptic to abandon H_1_ in favor of H_2_.

Second, the theorem in its original form does not apply to models with unknown parameters. Various extensions were made during the ensuing decades, among them Wilks’ (Wilks, 1938) and Wald’s (Wald, 1943) theorems. The Wilks-Wald extension allows the test of two composite models (models with one or more unknown parameters) in which one model, taken as the null hypothesis, is formed from the other model (the alternative) by placing one or more constraints on the parameters. An example is a normal (*μ*, *σ*^2^) distribution with both mean *μ* and variance *σ*^2^ unknown as the model for the alternative hypothesis H_2_, within which the null hypothesis model *f*_1_ constrains the mean to be a fixed known constant: *μ* = *μ*_1_. In such scenarios, the null model is “nested” within the alternative model, that is, the null is a special version of the alternative in which the parameters are restricted to a subset of the parameter space (set of all possible parameter values). Wilks’ (Wilks, 1938) and Wald’s (Wald, 1943) theorems together provide the asymptotic distribution of a function of the likelihood ratio under both the null and alternative hypotheses, with estimated parameters taken into account. The function is the familiar “generalized likelihood ratio statistic,” usually denoted *G*^2^, given by

(31) $$G^{2}=-2\;\text{log}\left({\hat{L}}_{1}/{\hat{L}}_{2}\right),$$

where ${\hat{L}}_{1}$ and ${\hat{L}}_{2}$ are the likelihood functions, respectively for models *f*_1_ and *f*_2_, with each likelihood maximized over all the unrestricted parameters in that model. The resulting parameter estimates, known as the maximum likelihood (ML) estimates, form a prominent part of frequentist statistics theory (Pawitan, 2001). Let *θ* be the vector of unknown parameters in model *f*_2_ formed by stacking subvectors *θ*_21_ and *θ*_22_. Likewise, let *θ* under the restricted model *f*_1_ be formed by stacking the subvectors *θ*_11_ and *θ*_12_, where *θ*_11_ is a vector of fixed, known constants (i.e., all values in *θ*_21_ are fixed) and *θ*_12_ is a vector of unknown parameters. Wald’s (1943) theorem (after some mathematical housekeeping: Stroud, 1972) gives the asymptotic distribution of *G*^2^ as a non-central chisquare(*ν*, *λ*) distribution, with degrees of freedom *ν* equal to the difference between the number of estimated parameters in *f*_2_ and the number of estimated parameters in *f*_1_, and non-centrality parameter *λ* being a statistical (Mahalanobis) distance between the true parameter values under H_2_ and their restricted versions under H_1_:

(32) $$\lambda =n{\left(\theta_{21}-\theta_{11}\right)}^{\prime }\Sigma^{-1}\left(\theta_{21}-\theta_{11}\right).$$

Here Σ is a matrix of expected log-likelihood derivatives (details in Severini, 2000). Technically the true values *θ*_21_ must be local to the restricted values *θ*_11_; the important aspects for the present are that *λ* increases with *n* as well as with the effect size represented by the distance (*θ*_21_ − *θ*_11_)′Σ^−1^ (*θ*_21_ − *θ*_11_). With the true parameters equal to their restricted values, that is with H_1_ governing the data production, the non-centrality parameter becomes zero, and Wald’s theorem collapses to Wilks’ theorem, which gives the asymptotic distribution of *G*^2^ under H_1_ to be an ordinary chisquare(*ν*) distribution. For linear statistical models in the normal distribution family (regression, analysis of variance, etc.), *G*^2^ boils down algebraically into monotone functions of statistics with exact (non-central and central) t- or F-distributions, and so the various statistical hypothesis tests can take advantage of exact distributions instead of asymptotic approximations.

The concept of a confidence interval or region for one or more unknown parameters follows from Neyman-Pearson hypothesis testing in the form of a region of parameter values for which hypothesis H_1_ would not be rejected at fixed error rate *α*. We remark further that although a vast amount of every day science relies on the Wilks-Wald extension of Neyman-Pearson testing (and confidence intervals), frequentist statistics theory prior to the 1970s had not provided much advice on what to do when the two models are not nested.

Certainly nowadays one could setup a model *f*_1_(*x*) as H_1_ in a hypothesis test against a non-overlapping model *f*_2_(*x*) taken as H_2_ and obtain the distributions of the generalized likelihood ratio under both models with simulation/bootstrapping.

Third, the Neyman-Pearson Lemma provides no guidance in the event of model misspecification. The theorem assumes that the data was generated under either H_1_ or H_2_. However, the “Type 3” error of basing inferences on an inadequate model family is widely acknowledged to be a serious (if not fatal) scientific drawback of the Neyman-Pearson framework (and parametric modeling in general, see Chatfield, 1995). Modern applied statistics rightly stresses rigorous checking of model adequacy with various diagnostic procedures, such as the standard battery of residual analyses in regression models. Deciding between two models based on diagnostic qualities has been a standard workaround in the situation mentioned above for which the two models are not nested. For instance, one might choose the model with the most homoscedastic residuals.

Fourth, the asymmetry of the error structure has led to difficulties in scientific interpretation of Neyman-Pearson hypothesis testing results. The difficulties stem from *α* being a fixed constant. A decision to prefer hypothesis H_2_ over H_1_ because the LR (Equation 23) is smaller than *c* is not so controversial. The H_2_ over H_1_ decision has some intuitively desirable statistical properties. For example, the error rate *β* asymptotically approaches 0 as the sample size *n* grows larger. Further, *β* asymptotically approaches 0 as model *f*_2_ becomes “farther” from *f*_1_ (in the sense of the symmetric KL distance *K*_12_ + *K*_21_ as seen in Equation 30). Mired in controversy and confusion, however, is the decision to prefer H_1_ over H_2_ when the LR is larger than *c*. The value of *c* is set by the chosen value of the error rate *α*, using the probabilistic properties of model *f*_1_. If a larger sample size is used, the LR has more terms, and the value of *c* necessary to attain the desired value of *α* changes. In other words, *c* depends on sample size *n* and moves in such a way as to keep *α* fixed (at 0.05 or whatever other value is used; Equation 28). The net effect is to leave the Neyman-Pearson framework without a mechanism to assess evidence *for* H_1_, for no matter how far apart the models are or how large a sample size is collected, the probability of wrongly choosing H_2_ when H_1_ is true remains stuck at *α*.

Fifth, scientific practice rarely stops with just two models. In an analysis of variance, after an overall test of whether the means are different, one usually needs to sort out just who is bigger than whom. In a multiple regression, one is typically interested in which subset of predictor variables provide the best model for predicting the response variable. In a categorical data analysis of a multiway contingency table, one is often seeking to identify which combination of categorical variables and lower and higher order interactions best account for the survey counts. For many years (through the 1980s at least), standard statistical practice called for multiple models to be sieved through some (often long) sequence of Neyman-Pearson tests, through processes such as multiple pairwise comparisons, stepwise regression, and so on. It has long been recognized, however, that selecting among multiple models with Neyman-Pearson tests plays havoc with error rates, and that a pairwise decision tree of “yes-no’s” might not lead to the best model among multiple models (Whittingham et al., 2006 and references therein). Using Neyman-Pearson tests for selection among multiple models was (admittedly among statisticians) a kludge to be used only until something better was developed.

#### 2.1.2. Fisher Significance Analysis

R. A. Fisher never fully bought into the Neyman-Pearson framework, although generations of readers have debated about what exactly Fisher was arguing for, due to the difficulty of his writing style and opacity of his mathematics. Fisher rejected the scientific usefulness of the alternative hypothesis (likely in part because of the lurking problem of misspecification) and chose to focus on single-model decisions (resulting in lifelong battles with Neyman; see the biography by Box, 1978). Yea or nay, is model *f*_1_ an adequate representation of the data? As in the Neyman-Pearson framework, Fisher typically cast the null hypothesis H_1_ in the role of a skeptic’s hypothesis (the lady cannot tell whether the milk or the tea was poured first). It was scientifically sufficient in this approach for the researcher to develop evidence to dissuade the skeptic of the adequacy of the null model. The inferential ambitions here are necessarily more limited, in that no alternative model is enlisted to contribute more insights for understanding the phenomenon under study, such as an estimate of effect size. As well, Fisher’s null hypothesis approach preserves the Neyman-Pearson incapacitation when the null model is not contradicted by data, in that at best, one will only be able to say that the data are a plausible realization of observations that could be generated under H_1_.

Fisher’s principal tool for the inference was the *P*-value. For Fisher’s preferred statistical distribution models, the data enter into the maximum likelihood estimate of a parameter in the form of a statistic, such as the sample mean. The implication is that such a statistic carries all the inferential information about the parameter; knowing the statistic’s value is the same (for purposes of inference about the parameter) as knowing the values of all the individual observations. Fisher coined the term “sufficient statistic” for such a quantity. The null model in Fisher significance analysis is formed by constraining a parameter to a pre-specified value. In the tea testing example, the probability of correct identification is constrained to one half. Fisher’s *P*-value is the probability that data drawn from the model H_1_ yield a sufficient statistic as extreme or more extreme than the sufficient statistic calculated from the real data.

In absence of an alternative model, Fisher’s strict *P*-value accomplishes an inference similar to what is called a goodness of fit test (or model adequacy test) in contemporary practice, as the inference seeks to establish whether or not the data plausibly could have arisen from model *f*_1_. Accordingly, just about any statistic (besides a sufficient statistic) can be used to obtain a *P*-value, provided the distribution of the statistic can be derived or approximated under the model *f*_1_. Goodness of fit tests therefore tend to multiply, as witnessed by the plethora of tests available for the normal distribution. To sort out the qualities of different goodness of fit tests, one usually has to revert to a Neyman-Pearson two-model framework to establish for what types of alternative models a particular test is powerful.

#### 2.1.3. Neyman-Pearson Testing With P-values

*P*-values are, of course, routinely used in Neyman-Pearson hypothesis testing, but the inference is different from that made with Fisher significance. A *P*-value in the context of the generalized LR test above (Equation 31) is defined as the probability that, if the data generation process were to be repeated, the new value of the LR would exceed the one already observed, provided that the data were generated under H_1_. Hinkley (1987) interprets the *P*-value as the Type 1 error rate that an ensemble of hypothetical experiments would have if their critical level *c* was set to the observation of this experiment. In the generalized LR test, the approximate *P*-value would simply be the area to the right of the observed value of *G*^2^ under the chisquare pdf applicable for H_1_-generated data. For Fisher’s preferred statistical distributions (those with sufficient statistics, nowadays called exponential family distributions), the generalized LR statistic *G*^2^ algebraically reduces to a monotone function of one or more sufficient statistics for the parameter or parameters under constraint in the model *f*_1_. In the generalized likelihood ratio framework, the hypothesis test decision between H_1_ and H_2_ can be made by comparing the *P*-value to the fixed value of *α*, rejecting H_1_ as a plausible origin of the data if the *P*-value is ≤ *α*.

In both Neyman-Pearson hypothesis testing and Fisher significance analysis, the *P*-value provides no evidence for model H_1_. The *P*-value in the two-model framework has been thought of as an inverse measure of the “evidence” for H_2_, as the distribution of the *P*-value under data generated by H_2_ becomes more and more concentrated near zero as sample size becomes large or as model *f*_2_ becomes “farther” from *f*_1_. In the Fisher one-model framework an alternative model is unspecified. Consequently, a low *P*-value has been interpreted as “evidence” against H_1_. However, the *P*-value under data generated by H_1_ has a uniform distribution (because a continuous random variable transformed by its own cumulative distribution function has a uniform distribution) no matter what the sample size is or how far away the true data generating process is. Hence, as with NP tests, Fisher’s *P*-value has no evidential value toward *f*_1_, as any *P*-value is equally likely under H_1_.

Ecologists use and discuss hypothesis testing in both the Fisher sense and the Neyman-Pearson sense, sometimes referring to both enterprises as “null hypothesis testing.” The use of *P*-values, strongly argued for by some (Hurlburt and Lombardi 2009), does not in and of itself distinguish the two approaches. Rather, a specific alternative hypothesis, an estimable effect size, and (most controversially) a decision rule fixing a Type 1 error rate (i.e., comparing a *P*-value to *α*) identifies the analysis as more Neyman-Pearsonian than Fisherian. While Fisher himself originated the *P* ≤ 0.05 tradition for judging whether a deviation is significant [… “it is convenient to draw the line at about the level at which we can say: ‘Either there is something in the treatment, or a coincidence has occurred, such as does not occur more than once in twenty trials.’” Fisher (1926)], he was mostly casual about the cutoff and viewed *P*-values more as evidence against the null hypothesis in question. In ecology, null hypotheses in the Fisherian sense are seen, for instance, in analyses of species assembly patterns in ecological communities, such as in testing whether bird species groups on o shore islands could be modeled as randomly drawn from the mainland (Connor and Simberlo, 1979). By contrast, a field experiment aimed at demonstrating the existence of competition and estimating an effect size (Underwood, 1986) would take on a Neyman-Pearsonian flavor.

#### 2.1.4. Equivalence Testing and Severity

Attempts have been made to modify the Neyman-Pearson framework to accommodate the concept of evidence for H_1_. In some applied scientific fields, for example in pharmacokinetics and environmental science, the regulatory practice has created a burden of proof around models normally regarded as null hypothesis models: the new drug has an effect equal to the standard drug, the density of a native plant has been restored to equal its previous level (Anderson and Hauck, 1983; McDonald and Erickson, 1994; Dixon, 1998). Equivalence testing and non-inferiority testing (e.g., Wellek, 2010) are statistical methods designed to address the problem that “absence of a significant effect” is not the same as “an effect is significantly absent.” In practice, the equivalence testing methods reverse the role of null and alternative hypotheses by specifying a parameter region that constitutes an acceptably small departure from the parameter’s constrained value and then casting the region as the alternative hypothesis. Typically, two statistical hypothesis tests are required to conclude that the parameter is within the small region containing the constraint, such as two one-sided *t*-tests (to show that the parameter is bounded by each end of the region).

Another proposed solution for the evidence-for-the-null-hypothesis problem is the concept of severity (Mayo, 1996, 2018; Mayo and Spanos, 2006) and the closely related method of reverse testing (Parkhurst, 2001). Severity is a sort of *P*-value under a specified (or possibly estimated) version of the alternative hypothesis. It is the probability that a test statistic more extreme than the one observed would be obtained if the experiment were to be repeated, if the data were arising from model *f*_2_ (with the particular effect size specified). In the generalized likelihood ratio framework, the severity would be calculated as the area to the right of the observed value of *G*^2^ under the non-central chisquare pdf applicable for data generated under model *f*_2_, with the non-centrality parameter set at a specified value. Thus, severity is a kind of attained power for a particular effect size. Also, severity is mostly discussed in connection with one-sided hypotheses, so that its calculation under the two-sided generalized likelihood ratio statistic is at best an approximation. However, if the effect size is substantial, the probability contribution from the “other side” is low, and the approximation is likely to be fine. In general, the severity of the test is related to the size of the effect, so care needs to be taken in the interpretation of the test.

For a given value of the LR, if the effect size is high, the probability of obtaining stronger evidence against H_1_ is high, and the severity of the test against H_1_ is high. “A claim is severely tested to the extent that it has been subjected to and passes a test that probably would have found flaws, were they present” (Mayo, 2018).

For both equivalence testing and severity, we are given procedures in which consideration of evidence requires two statistics and analyses. In the case of equivalence testing, we have a statistical test for each side of the statistical model specified by H_1_, and for severity we have a statistic for H_2_ and a statistic for H_1_. Indeed, Thompson (2007), section 11.2, considers that for *P*-values to be used as evidence for one model over another, these must be used in pairs. There is evidence for H_1_ relative to H_2_ if the first *P*-value, say *P*_1_, is large and the second *P*-value, say *P*_2_, is small. The requirement for two analyses and two interpretations seems a disadvantageous burden for applications. More importantly, the equivalence testing and severity concepts do not yet accommodate the problems of multiple models or non-nested models.

#### 2.1.5. Royall’s Concept of Evidence

The LR statistic (Equation 7), as discussed by Hacking (1965) and Edwards (1972), can be regarded as a measure of *evidence* for H_1_ and against H_2_ (Edwards 1972 termed it *support*, but the word has a different technical meaning in probability and is better avoided here), or equivalently, an inverse measure of evidence for H_2_ and against H_1_. The evidence concept here is post-data in that the realized value of the LR itself, and not a probability calculated over hypothetical experiment repetitions, conveys the magnitude of the empirical scientific case for H_1_ or H_2_. However, restricting attention to just the LR itself leaves the prospect of committing an error unanalyzed; while scientists want to search for truth, they strongly want (for reasons partly sociological) to avoid being wrong.

Royall (1997, 2000) argued forcefully for greater use of evidence-based inferences in statistics, and to Hacking’s and Edwards’ frameworks he added formal procedures and consideration of errors. Royall’s basic setup uses completely specified models as in Neyman-Pearson, but the conclusion about which model is favored by the data is based on fixed thresholds for the LR value, not thresholds determined by any error rate. The idea is to conclude there is strong evidence in favor of model H_1_ when *L*_1_ is *k* times *L*_2_ and strong evidence in favor of H_2_ when *L*_2_ is *k* times *L*_1_. Royall’s conclusion structure in terms of the LR then has a trichotomy of outcomes:

(33) $$L_{1}/L_{2}\geq k:\;\text{Strong evidence for}\;{\text{H}}_{1}.\;1/k<L_{1}/L_{2}<k:\;\text{Weak or inconclusive evidence}.\;L_{1}/L_{2}\leq 1/k:\;{\text{ Strong evidence for H}}_{2}.$$

For *k*, values of 8, 20, or 32 are mentioned. The *k* value is chosen by the investigator, but unlike *α* in the Neyman-Pearson framework, *k* is not dependent on sample size. Viewed as evidence, LR is a post-data measure. The inference does not make appeals to hypothetical repeated sampling.

Royall (1997, 2000) moreover defines pre-data error rates which are potentially useful in experimental design and serve as reassurance that the evidential approach will not lead investigators astray too often. Suppose the data were generated by model *f*_1_. It is possible that the LR could take a wayward value, leading to one of two possible errors in conclusion that could occur: (1) the LR could take a value corresponding to weak or inconclusive evidence (the error of weak evidence), or (2) the LR could take a value corresponding to strong evidence for H_2_ (the error of misleading evidence). Given the data are generated by model *f*_1_, the probabilities of the two possible errors are defined as follows:

(34) $$\text{P}\left(\text{ weak evidence }|{\text{H}}_{1}\right)=\text{P}\left(1/k<L_{1}/L_{2}<k|{\text{H}}_{1}\right)=W_{1}$$

(35) $$\text{P}\left(\text{ misleading evidence }|{\text{H}}_{1}\right)=\text{P}\left(L_{1}/L_{2}\leq 1/k|{\text{H}}_{1}\right)=M_{1}.$$

Similarly, given the data are generated under H_2_,

(36) $$\text{P}\left(\text{ weak evidence }|{\text{H}}_{2}\right)=\text{P}\left(1/k<L_{1}/L_{2}<k|{\text{H}}_{2}\right)=W_{2},$$

(37) $$\text{P}\left(\text{ misleading evidence }|{\text{H}}_{2}\right)=\text{P}\left(L_{1}/L_{2}\geq k|{\text{H}}_{2}\right)=M_{2}.$$

The error probabilities *M*_1_, *M*_2_, *W*_1_, and *W*_2_ can be approximated with the CLT results for *L*_1_/*L*_2_ (Equations 11–16). Proceeding as before with the Neyman-Pearson error rates, we find that

(38) $$M_{1}\approx \Phi \left(-\frac{\sqrt{n}}{\sigma_{1}}\left[\frac{1}{n}\text{log}(k)+K_{12}\right]\right),$$

(39) $$M_{2}\approx \Phi \left(-\frac{\sqrt{n}}{\sigma_{2}}\left[\frac{1}{n}\text{log}(k)+K_{21}\right]\right),$$

(40) $$W_{1}\approx \Phi \left(\frac{\sqrt{n}}{\sigma_{1}}\left[\frac{1}{n}\text{log}(k)-K_{12}\right]\right)\;-\Phi \left(-\frac{\sqrt{n}}{\sigma_{1}}\left[\frac{1}{n}\text{log}(k)+K_{12}\right]\right),$$

(41) $$W_{2}\approx \Phi \left(\frac{\sqrt{n}}{\sigma_{2}}\left[\frac{1}{n}\text{log}(k)-K_{21}\right]\right)\;-\Phi \left(-\frac{\sqrt{n}}{\sigma_{2}}\left[\frac{1}{n}\text{log}(k)+K_{21}\right]\right).$$

The error probabilities *M*_1_, *M*_2_, *W*_1_, and *W*_2_ depend on the models being compared, but it is easy to show that all four probabilities, as approximated by Equations (38–41), converge to zero as sample size *n* becomes large. For either hypothesis H_*i*_ (*i* = 1, 2), the total error probability given by *M*_*i*_ + *W*_*i*_ is additionally a monotone decreasing function of *n*, as for instance

(42) $$M_{1}+W_{1}=\Phi \left(\frac{\sqrt{n}}{\sigma_{1}}\left[\frac{1}{n}\text{log}(k)-K_{12}\right]\right),$$

in which the argument of the cdf Φ (_■_) is seen (by ordinary differentiation, assuming *k* > 1) to be monotone decreasing in *n* (the expression for *M*_2_ + *W*_2_ would have *σ*_2_ and *K*_21_ in place of *σ*_1_ and *K*_12_).

The probability *V*_1_ of strong evidence for model *f*_1_ (*x*), given the data are indeed generated by model *f*_1_ (*x*), becomes

(43) $${\text{V}}_{1}=1-M_{1}-W_{1},$$

with *V*_2_ = 1 − *M*_2_ − *W*_2_ defined in kind. Here *V* stands for veracity or veridicality (because of context, there should be no confusion with the variance operator). It follows from the monotone property of *M*_*i*_ + *W*_*i*_ that *V*_*i*_ is a monotone increasing function of *n*. Furthermore, it is straightforward to show that *V*_*i*_ > *M*_*i*_, *i* = 1, 2.

Note that *V*_1_, *M*_1_, and *W*_1_ are not in general equal to their counterparts *V*_2_, *M*_2_, and *W*_2_, nor should we expect them to be; frequencies of errors will depend on the details of the model generating the data. One model distribution with, say, a heavy tail could produce errors at a greater rate than a light-tailed model. The asymmetry of errors suggests possibilities of pre-data design to control errors. For instance, instead of LR cutoff points 1/*k* and *k*, one could find and use cutoff values *k*_1_ and *k*_2_ that render M_1_ and M_2_ nearly equal for a particular sample size and particular values of *σ*_1_, *K*_12_, *σ*_2_, and *K*_21_. Such design, however, will induce an asymmetry in the error rates (defined below) for misspecified models.

Interestingly, as a function of *n*, *M*_*i*_ (*i* = 1, 2) increases at first, rising to a maximum value before decreasing asymptotically to zero. The value *ñ*_1_ at which *M*_1_ is maximized is found by maximizing the argument of the normal cdf in Equation (38):

(44) $${\tilde{n}}_{1}=\frac{\text{log}(k)}{K_{12}},$$

with the corresponding maximum value of *M*_1_ being

(45) $${\tilde{M}}_{1}=\Phi \left(-\frac{2\sqrt{K_{12}\text{log}(k)}}{\sigma_{1}}\right).$$

Expressions for *ñ*_2_ and ${\tilde{M}}_{2}$ are similar and substitute *K*_21_ and *σ*_2_ in place of the H_1_ quantities. That the *M*_*i*_ functions would increase with *n* initially is counterintuitive at first glance. With just a few observations, the variability of the likelihood ratio is not big enough to provide much chance of misleading evidence, although the chance of weak evidence is high. As the sample size increases, the chance of misleading evidence grows at first, replacing some of the chance of weak evidence, before decreasing. It is the overall probability of either weak or misleading evidence, *W*_*i*_ + *M*_*i*_, that decreases monotonically with sample size.

#### 2.1.6. Illustration of the Concept of Evidence

We illustrate the error properties of evidence under correct model specification with an example. Suppose the values *x*_1_, *x*_2_, … , *x*_*n*_ are zeros and ones that arose as iid observations from a Bernoulli distribution with *P*(*X* = 1) = *p*. The pdf is *f* (*x*) = *p*^*x*^(1 − *p*)^1−*x*^, where *x* is 0 or= 1. The sum of the observations of course has a binomial distribution. We wish to compare hypothesis H_1_: *p* = *p*_1_ with H_2_: *p* = *p*_2_, where *p*_1_ and *p*_2_ are specified values. The log-likelihood ratio is

(46) $$\text{log}\left(\frac{L_{1}}{L_{2}}\right)=\left(\overset{n}{\underset{i=1}{\sum }}x_{i}\right)\text{log}\left(\frac{p_{1}}{p_{2}}\right)+\left(n-\overset{n}{\underset{i=1}{\sum }}x_{i}\right)\text{log}\left(\frac{1-p_{1}}{1-p_{2}}\right)$$

From Equations (4) and (9) we find that

(47) $$K_{12}=p_{1}\text{log}\left(\frac{p_{1}}{p_{2}}\right)+\left(1-p_{1}\right)\text{log}\left(\frac{1-p_{1}}{1-p_{2}}\right),$$

(48) $${\sigma_{1}}^{2}=p_{1}{\left[\text{log}\left(\frac{p_{1}}{p_{2}}\right)\right]}^{2}+\left(1-p_{1}\right){\left[\text{log}\left(\frac{1-p_{1}}{1-p_{2}}\right)\right]}^{2}-{K_{12}}^{2}.$$

**FIGURE 3 |:**
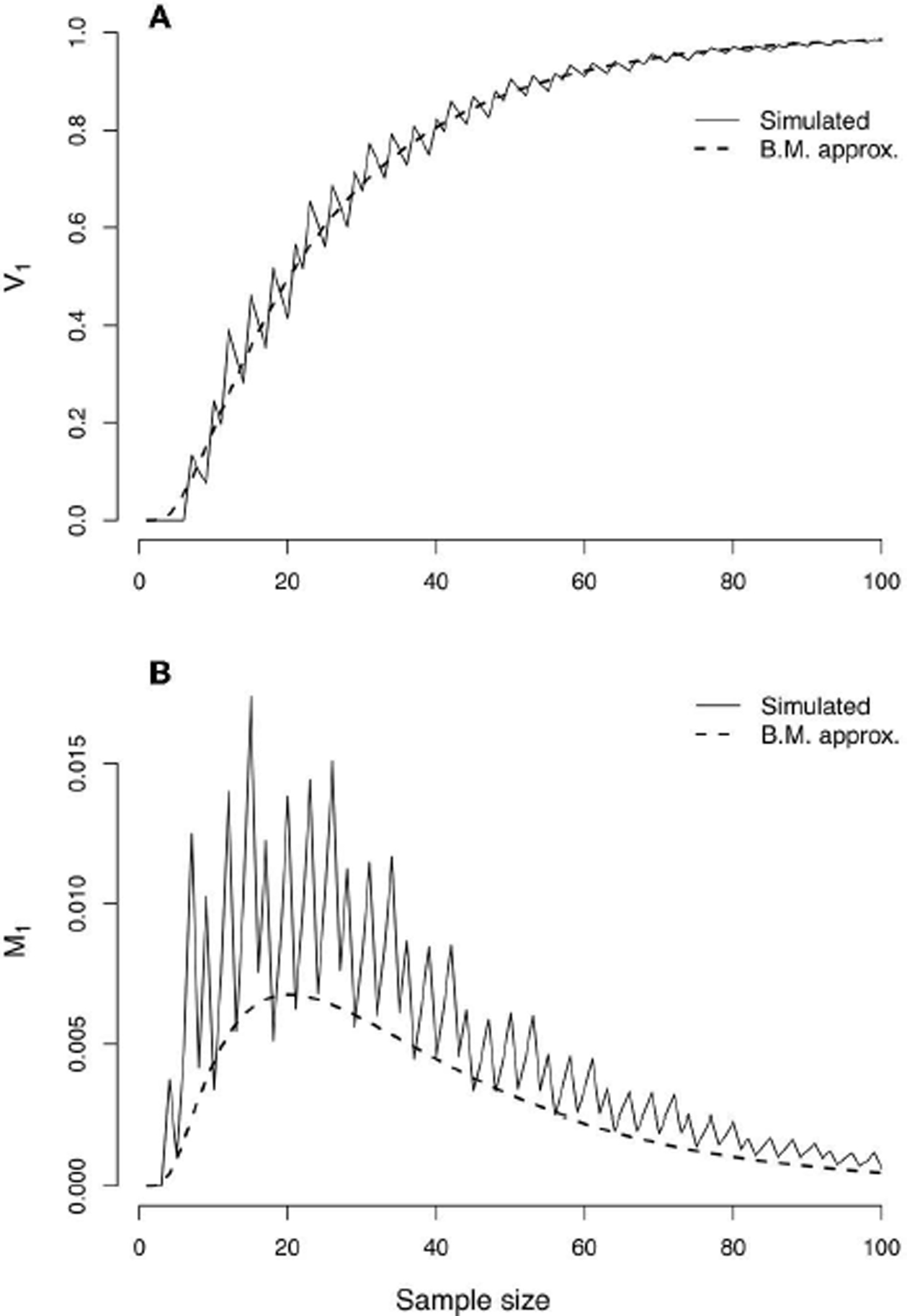
Evidence error probabilities for comparing two Bernoulli(*p*) distributions, with *p*_1_ = 0.75 and *p*_2_ = 0.50. **(A)** Simulated values (jagged curve) and values approximated under the Central Limit Theorem of the probability of strong evidence for model *H*_1_, *V*_1_ = 1 − *M*_1_ − *W*_1_. **(B)** Simulated values (jagged curve) and approximated values for the probability of misleading evidence *M*_1_. Note that the scale of the bottom graph is one fifth of that of the top graph.

In the top panel of Figure 3, simulated values of the probability of strong evidence for model *H*_1_, given by *V*_1_ = 1 − *M*_1_ − *W*_1_, are compared with the values as approximated with the CLT (Equations 38, 40). The simulated values create a jagged curve due to the discrete nature of the Bernoulli distribution but are well-characterized by the CLT approximation. The lower panel of Figure 3 portrays the probability of misleading evidence given by *M*_1_ as a function of *n*. The discrete serrations are even more pronounced in the simulated values of *M*_1_, and the CLT approximation (Equation 38) follows only the lower edges; the approximation could likely be improved (i.e., set toward the middle of the serrated highs and lows) with a continuity correction. The CLT nonetheless picks up the qualitative behavior of the functional form of *M*_1_.

#### 2.1.7. P-values, Severity, and Evidence

The concept of evidence allows re-interpretation of *P*-values in a clarifying manner. Suppose we denote by *l*_1_/*l*_2_ the realized (i.e., post-data) value of the LR, the lower case signaling the actual outcome rather than the random variable (pre-data) version of the LR denoted by *L*_1_/*L*_2_. The classical *P*-value is the probability, given the data arise from model H_1_, that a repeat of the experiment would yield a LR value more extreme than the value *l*_1_/*l*_2_ that was observed. In our CLT setup, we can write

(49) $$P=\text{P}\left(\frac{L_{1}}{L_{2}}\leq \frac{l_{1}}{l_{2}}|{\text{H}}_{1}\right)\approx \Phi \left(-\frac{\sqrt{n}}{\sigma_{1}}\left[\frac{1}{n}\text{log}\left(\frac{l_{2}}{l_{1}}\right)+K_{12}\right]\right).$$

Comparing *P* with the expression for *M*_1_ (Equation 38), we find that *P* is the probability of misleading evidence under model *f*_1_ if the experiment were repeated and the value of *k* were taken as *l*_2_/*l*_1_.

If the value of *l*_1_/*l*_2_ is considered to be the evidence provided by the experiment, the value of *P* is a monotone function of *l*_1_/*l*_2_ and thereby might be considered to be an evidence measure on another scale. *P* however is seen to depend on other quantities as well: for a given value of *l*_1_/*l*_2_, *P* could be greater or less depending on the quantities *n*, *K*_12_, and *σ*_1_. Furthermore, *K*_21_ and *σ*_2_ are left out of the value of *P*, giving undue influence to model *f*_1_ in the determination of amount of evidence, a finger on the scale so to speak. The evidential framework therefore argues for the following distinction in the interpretation of *P*: the *evidence* is *l*_1_/*l*_2_, while *P*, like *M*_1_, is a probability of misleading evidence, except that *P* is defined post-data.

In fairness to both models, we can define two *P*-values based on the extremeness of evidence under model *f*_1_ and under model *f*_2_:

(50) $$P_{1}=P\left(\frac{L_{1}}{L_{2}}\leq \frac{l_{1}}{l_{2}}|H_{1}\right)\approx \Phi \left(-\frac{\sqrt{n}}{\sigma_{1}}\left[\frac{1}{n}\text{log}\left(\frac{l_{2}}{l_{1}}\right)+K_{12}\right]\right),$$

(51) $$P_{2}=P\left(\frac{L_{1}}{L_{2}}\leq \frac{l_{2}}{l_{1}}|H_{2}\right)\approx \Phi \left(-\frac{\sqrt{n}}{\sigma_{2}}\left[\frac{1}{n}\text{log}\left(\frac{l_{2}}{l_{1}}\right)+K_{21}\right]\right).$$

These are interpreted as the probabilities of misleading evidence under models 1 and 2, respectively if the value of *k* were taken to be *l*_2_/*l*_1_. The quantity 1 − *P*_2_ in this context is the severity as defined by Mayo (1996, 2018) and Mayo and Spanos (2006). Taper and Lele (2011) termed *P*_1_ or *P*_2_ as a local probability of misleading evidence (*M*_L_ in their notation), as opposed to a global, pre-data probability of misleading evidence (*M*_G_ in their notation; *M*_1_ and *M*_2_ here) characterizing the long-range reliability of the design of the data-generating process.

### 2.2. Misspecified Models

George Box’s (Box, 1979) oft-quoted aphorism that “all models are wrong, but some are useful” becomes pressing in ecology, a science in which daily work and journal articles are filled with statistical and mathematical representations. Ecologists must assume in abundance that Type 3 errors are prevalent, even routine, in their work. Here we compare Neyman-Pearson hypothesis testing with evidential statistics to try to understand how analyses can go wrong, and how analyses can be made better, in ecological statistics. For a statistical method of choosing between *f*_1_ (*x*) or *f*_2_ (*x*), we now ask how well the method performs toward choosing the model closest to the true model *g* (*x*) when both candidate models are misspecified.

#### 2.2.1. Neyman-Pearson Hypothesis Testing Under Misspecification

Statisticians have long cautioned about the prospect that both models *f*_1_ and *f*_2_ in the Neyman-Pearson framework, broadly interpreted to include testing composite models with generalized likelihood ratio and other approaches, could be misspecified, and as a result that the advertised error rates (or by extension the coverage rates for confidence intervals) would become distorted in unknown ways (for instance, Chatfield, 1995). The approximate behavior of the LR under the CLT under misspecification (Equations 20–22) allows us to view directly how the error probabilities *α* and *β* can be a ected in Neyman-Pearson testing when the models are misspecified.

The critical value *c* (Equation 28) is chosen as before, under the assumption that the observations are generated from model *f*_1_. We ask the following question: “Suppose the real Type 1 error is defined as picking model *f*_2_ when the model *f*_1_ is actually closest to the true pdf *g* (*x*) (that is, when Δ*K* > 0). What is the probability, let us say *α*′, of this Type 1 error, given that *f*_1_ is the better model?” We now have

(52) $$\frac{L_{1}}{L_{2}}\leq c\Rightarrow \frac{\sqrt{n}}{\sigma_{g}}\left[\frac{1}{n}\text{log}\left(\frac{L_{1}}{L_{2}}\right)-\Delta K\right]\leq \frac{\sqrt{n}}{\sigma_{g}}\left[\frac{1}{n}\text{log}(c)-\Delta K\right]\;=\frac{\sqrt{n}}{\sigma_{g}}\left(K_{12}-\Delta K\right)-\frac{\sigma_{1}}{\sigma_{g}}z_{\alpha }$$

after substituting for *c* (Equation 28), and so the CLT (Equation 22) tells us that

(53) $$\alpha^{\prime }=\text{P}\left(\frac{L_{1}}{L_{2}}\leq c|\Delta K>0\right)\approx \Phi \left(\frac{\sqrt{n}}{\sigma_{g}}\left(K_{12}-\Delta K\right)-\frac{\sigma_{1}}{\sigma_{g}}z_{\alpha }\right)\;\neq \Phi \left(-z_{\alpha }\right)=\alpha .$$

In words, the Type 1 error realized under model misspecification is generally not equal to the specified test size. Note that Equation (53) collapses to Equation (28), as desired, if *f*_1_ = *g*.

Whether the actual Type 1 error probability *α*′ is greater than, equal to, or less than the advertised level *α* depends on the various quantities arising from the configuration of *f*_1_ (*x*), *f*_2_ (*x*), and *g* (*x*) in model space. Because the standard normal cdf Φ (_■_) is a monotone increasing function, we have

(54) $$\alpha^{\prime }>\alpha \Rightarrow \frac{\sqrt{n}}{\sigma_{g}}\left(K_{12}-\Delta K\right)-\frac{\sigma_{1}}{\sigma_{g}}z_{\alpha }>-z_{\alpha }.$$

The inequality reduces to three cases, depending on whether *σ*_1_ − *σ*_*g*_ is positive, zero, or negative:

(55) $$\alpha^{\prime }>\alpha \Rightarrow \;\sqrt{n}\frac{\left(K_{12}-\Delta K\right)}{\left(\sigma_{1}-\sigma_{g}\right)}>z_{\alpha },\text{ if }\sigma_{1}-\sigma_{g}>0,$$

(56) $$K_{12}-\Delta K>0,\text{ if }\sigma_{1}-\sigma_{g}=0,$$

(57) $$\sqrt{n}\frac{\left(K_{12}-\Delta K\right)}{\left(\sigma_{1}-\sigma_{g}\right)}<z_{\alpha },\text{ if }\sigma_{1}-\sigma_{g}<0.$$

The ratio (K_12_ − Δ*K*) / (*σ*_1_ − *σ*_*g*_) compares the difference between what we assumed about the LR mean (*K*_12_) and what is the actual mean (Δ*K*) with the difference between what we assumed about the LR variability (*σ*_1_) with what is the actual variability (*σ*_*g*_). The left-hand inequalities for each case are reversed if *α*′ < *α*.

The persuasive strength of Neyman-Pearson testing always revolved around the error rate *α* being known and small, and the *P*-value, if used, being an accurate reflection of the probability of more extreme data under H_1_. When *L*_1_/*L*_2_ ≤ *c* in the Neyman-Pearson framework with correctly specified models, the reasoned observer is forced to abandon H_1_ as untenable. However, in the presence of misspecification, the real error rate *α*′ is unknown, as is a real *P*-value for a generalized likelihood ratio test. Furthermore, *α*′ is seen in Equation (53) to be an increasing function of *n* if *K*_12_ > Δ*K* (remember that for a generalized LR test the Type 1 error is predicated on Δ*K* > 0), *with 1 as an upper asymptote*. If model *f*_2_ is very different from model *f*_1_ (*K*_12_ large) but is almost as close to truth as *f*_1_ (Δ*K* small), then Type 1 errors will be rampant, more so with increasing sample size.

That greater sample size would make error more likely seems counterintuitive, but it can be understood from the CLT results for the average log-LR given by (1/*n*) log (*L*_1_/*L*_2_) (Equations 12, 21). If the observations arise from *f*_1_ (*x*) (correct specification), the average log-LR has a mean of *K*_12_ and its distribution becomes more and more concentrated around *K*_12_ as *n* becomes large. If however the observations arise from *g* (*x*) (misspecification), the average log-LR has a mean of Δ*K* and its distribution becomes more and more concentrated around Δ*K* as *n* becomes large. A Neyman-Pearson test based on a statistic that has a null hypothesis mean of *K*_12_ will become more and more certain to reject the null hypothesis when the true mean is Δ*K*. Thus, the Neyman-Pearson framework can be a highly unreliable approach for picking the best model in the presence of misspecification.

The error probability *β*′ is defined and approximated in similar fashion. If model *f*_2_ is closer to truth, we have Δ*K* < 0, and from Equations (28–30) we now have

(58) $$\frac{L_{1}}{L_{2}}>c\Rightarrow \frac{\sqrt{n}}{\sigma_{g}}\left[\frac{1}{n}\text{log}\left(\frac{L_{1}}{L_{2}}\right)-\Delta K\right]>\frac{\sqrt{n}}{\sigma_{g}}\left(K_{12}-\Delta K\right)-\frac{\sigma_{1}}{\sigma_{g}}z_{\alpha }.$$

The CLT then gives

(59) $$\beta^{\prime }=\text{P}\left(\frac{L_{1}}{L_{2}}>c|\Delta K<0\right)\;\approx 1-\Phi \left(\frac{\sqrt{n}}{\sigma_{g}}\left(K_{12}-\Delta K\right)-\frac{\sigma_{1}}{\sigma_{g}}z_{\alpha }\right)\;\neq 1-\Phi \left(\frac{\sqrt{n}}{\sigma_{2}}\left(K_{12}+K_{21}\right)-\frac{\sigma_{1}}{\sigma_{2}}z_{\alpha }\right)=\beta .$$

As a function of *n*, *β*′ goes to zero as *n* becomes large, preserving that desirable property of *β* from Neyman-Pearson testing under correct specification. However, if the experiment or survey is being planned around the value of *β*, under misspecification the actual value as defined by *β*′ could be quite different. In particular, if *β*′ > *β*, we must have

(60) $$\frac{\sqrt{n}}{\sigma_{g}}\left(K_{12}-\Delta K\right)-\frac{\sigma_{1}}{\sigma_{g}}z_{\alpha }<\frac{\sqrt{n}}{\sigma_{2}}\left(K_{12}+K_{21}\right)-\frac{\sigma_{1}}{\sigma_{2}}z_{\alpha }.$$

The inequality reduces to three cases depending on whether *σ*_2_ − *σ*_*g*_ is positive, zero, or negative:

(61) $$\beta^{\prime }>\beta \Rightarrow \;\frac{\sqrt{n}}{\sigma_{1}}\left[\frac{\sigma_{2}\left(K_{12}-\Delta K\right)-\sigma_{g}\left(K_{12}+K_{21}\right)}{\sigma_{2}-\sigma_{g}}\right]<z_{\alpha },\text{ if }\sigma_{2}-\sigma_{g}>0,$$

(62) $$(-\Delta K)-K_{21}<0,\text{ if }\sigma_{2}-\sigma_{g}=0,$$

(63) $$\frac{\sqrt{n}}{\sigma_{1}}\left[\frac{\sigma_{2}\left(K_{12}-\Delta K\right)-\sigma_{g}\left(K_{12}+K_{21}\right)}{\sigma_{2}-\sigma_{g}}\right]>z_{\alpha },\text{ if }\sigma_{2}-\sigma_{g}<0.$$

The left inequalities for the three cases are reversed for *β*′ < *β*. The degree to which *β*′ departs from *β* is seen to depend on a tangled bank of quantities arising from the configuration of *f*_1_ (*x*), *f*_2_ (*x*), and *g* (*x*) in model space.

#### 2.2.2. P-values, Equivalence Testing, and Severity Under Misspecification

The problems with *α* and *β*, and with *P*-values as defined for the generalized LR setting in Equations (50) and (51), under misspecification highlight problems that might arise in significance testing, equivalence testing or severity analysis. With misspecification, the true *P*-value (*P*′ say) can differ greatly from the *P*-value (Equation 49) calculated under H_1_ and thereby could promote misleading conclusions (*P*′ is formed from Equation (49) by substituting *σ*_*g*_ for *σ*_1_ and −Δ*K* for *K*_12_). Equivalence testing, being retargeted hypothesis testing, will take on all the problems of hypothesis testing under misspecification. Severity is 1 − *P*_2_ as defined by Equation (51), but with misspecification the true value of *P*_2_ is Equation (51) with *σ*_*g*_ substituted for *σ*_2_ and −Δ*K* substituted for *K*_21_. With misspecification, the true severity could differ greatly from the severity calculated under H_2_. One might reject H_1_ falsely, or one might fail to reject H_1_ falsely, or one might fail to reject H_1_ and falsely deem it to be severely tested. Certainly, in equivalence testing and severity analysis, the problem of model misspecification is acknowledged as important (for instance, Mayo and Spanos, 2006; Spanos, 2010) and is addressed with model evaluation techniques, such as residual analysis and goodness of fit testing.

#### 2.2.3. Evidence Under Misspecification

To study the properties of evidence statistics under model misspecification, we redefine the probabilities of weak evidence and misleading evidence in a manner similar to how the error probabilities were handled above in the Neyman-Pearson formulation. We take *W*_1_′ and *M*_1_′ to be the probabilities of weak and misleading evidence, given that model *f*_1_ is closer to truth, that is, given that Δ *K* > 0:

(64) $$\text{P}(\text{ weak evidence }|\Delta K>0)=\text{P}\left(1/k<L_{1}/L_{2}<k|\Delta K>0\right)={W_{1}}^{\prime },$$

(65) $$\text{P}(\text{ misleading evidence }|\Delta K>0)=\text{P}\left(L_{1}/L_{2}\leq 1/k|\Delta K>0\right)={M_{1}}^{\prime }.$$

Similarly, given model *f*_2_ is closer to truth,

(66) $$\text{P}(\text{ weak evidence }|\Delta K<0)=\text{P}\left(1/k<L_{1}/L_{2}<k|\Delta K<0\right)={W_{1}}^{\prime },$$

(67) $$\text{P}(\text{ misleading evidence }|\Delta K<0)=\text{P}\left(L_{1}/L_{2}\geq k|\Delta K<0\right)={M_{1}}^{\prime }.$$

The error probabilities *M*_1_′, *M*_2_′, *W*_1_′, and *W*_2_′ can be approximated with the CLT results for *L*_1_/*L*_2_ (Equations 20–22) under misspecification. For example, to approximate *M*_1_′ we note that

(68) $$\frac{L_{1}}{L_{2}}\leq \frac{1}{k}\Rightarrow \frac{\sqrt{n}}{\sigma_{g}}\left[\frac{1}{n}\text{log}\left(\frac{L_{1}}{L_{2}}\right)-\Delta K\right]\leq \frac{\sqrt{n}}{\sigma_{g}}\left[\frac{1}{n}\text{log}\left(\frac{1}{k}\right)-\Delta K\right]\;=-\frac{\sqrt{n}}{\sigma_{g}}\left[\frac{1}{n}\text{log}(k)+\Delta K\right].$$

We thus obtain

(69) $${M_{1}}^{\prime }\approx \Phi \left(-\frac{\sqrt{n}}{\sigma_{g}}\left[\frac{1}{n}\text{log}(k)+\Delta K\right]\right).$$

The other error probability under misspecification, with Δ*K* < 0, likewise becomes

(70) $${M_{2}}^{\prime }\approx \Phi \left(-\frac{\sqrt{n}}{\sigma_{g}}\left[\frac{1}{n}\text{log}(k)+\left|\Delta K\right|\right]\right).$$

The expression is identical to Equation (69) where Δ*K* > 0 and so we may write

(71) $${M_{i}}^{\prime }\approx \Phi \left(-\frac{\sqrt{n}}{\sigma_{g}}\left[\frac{1}{n}\text{log}(k)+\left|\Delta K\right|\right]\right),\;i=1,2.$$

In words, for models with no unknown parameters under misspecification, the error probabilities *M*_1_′ and *M*_2_′ are identical. Using different LR cutoff points *k*_1_ and *k*_2_ to control error probabilities *M*_1_ and *M*_2_ under correct specification would break the symmetry of errors under misspecification. The consideration of evidential error probabilities in study design forces the investigator to focus on what types of errors and possible model misspecifications are most important to the study.

The symmetry of error rates is preserved for weak evidence, for which we obtain

(72) $${W_{i}}^{\prime }\approx \Phi \left(\frac{\sqrt{n}}{\sigma_{g}}\left[\frac{1}{n}\text{log}(k)-\left|\Delta K\right|\right]\right)\;-\Phi \left(-\frac{\sqrt{n}}{\sigma_{g}}\left[\frac{1}{n}\text{log}(k)+\left|\Delta K\right|\right]\right),\;i=1,2.$$

**FIGURE 4 |:**
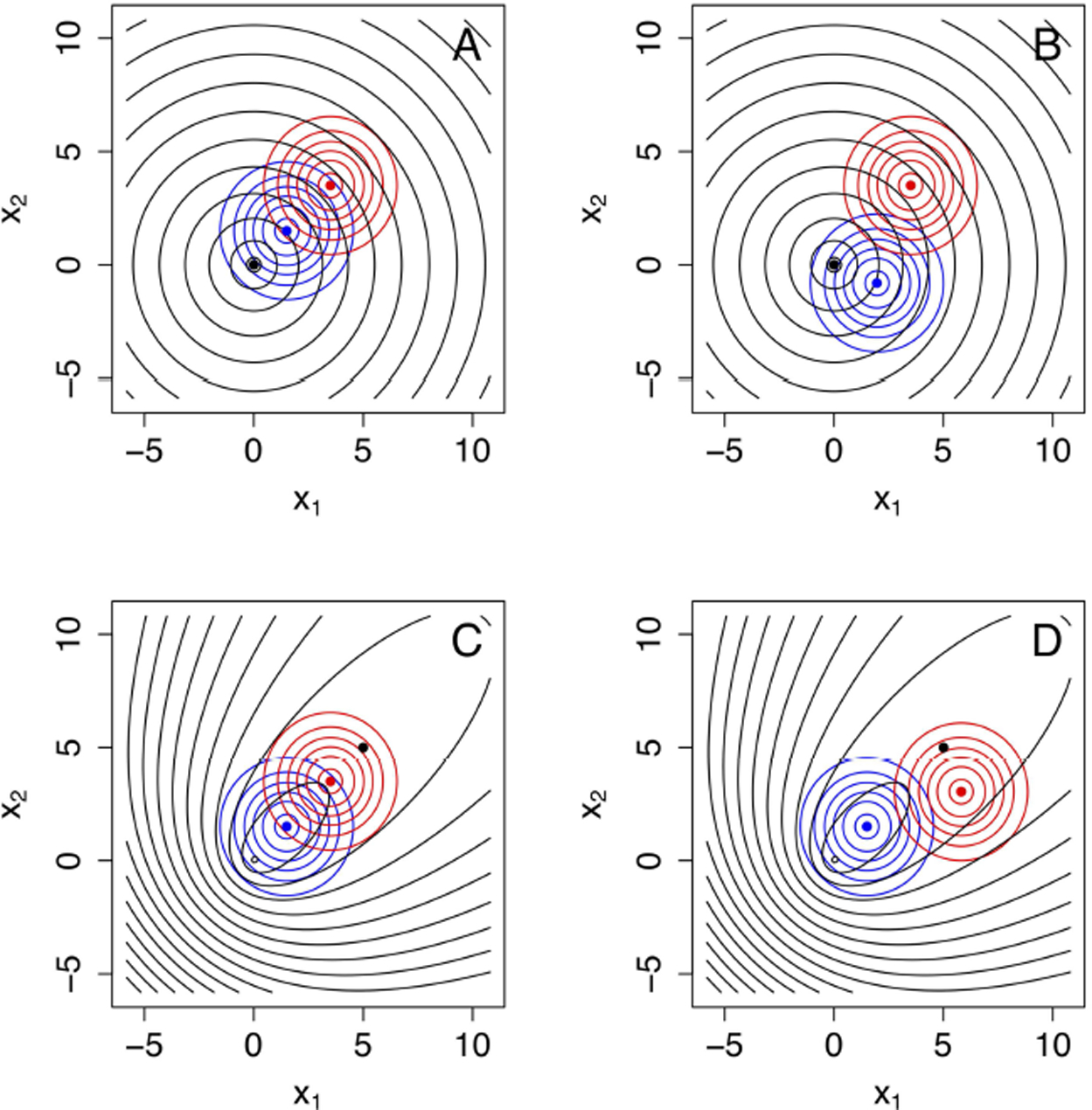
Four model configurations involving a bivariate generating process *g*(*x*_1_, *x*_2_) (in black), and two approximating models *f*_1_(*x*_1_, *x*_2_) (in blue) and *f*_2_(*x*_1_, *x*_2_) (in red). In all cases the approximating models are bivariate normal distributions whereas the generating process is a bivariate Laplace distribution. These model configurations are useful to explore changes in *α*′ (Equation 53), *β*′ (Equation 59) and $M_{i}^{\prime },W_{i^{\prime }},i=1,2$ (Equations 71, 72) as a function of sample size, as plotted in Figure 6. **(A)** *g*(*x*_1_, *x*_2_) is a bivariate Laplace distribution centered at 0 with high variance. All three models have means aligned along the 1: 1 line and marked with a black, blue, and red filled circle, respectively. Model *f*_1_(*x*_1_, *x*_2_) is closest to the generating process. **(B)** Model *f*_1_(*x*_1_, *x*_2_) is still the model closest to the generating process, at exactly the same distance as in **(A)** but misaligned from the 1: 1 line. **(C)** Here all three models are again aligned, but the generating process *g*(*x*_1_, *x*_2_) is an asymmetric bivariate Laplace that has a large mode at 0, 0 and smaller mode around the mean, marked with a black dot. In this case, the generating model is closer to model *f*_2_(*x*_1_, *x*_2_) (in red). **(D)** Same as in **(C)**, except model *f*_2_(*x*_1_, *x*_2_) (in blue) is now misaligned, but still the closest model to the generating process.

**FIGURE 5 |:**
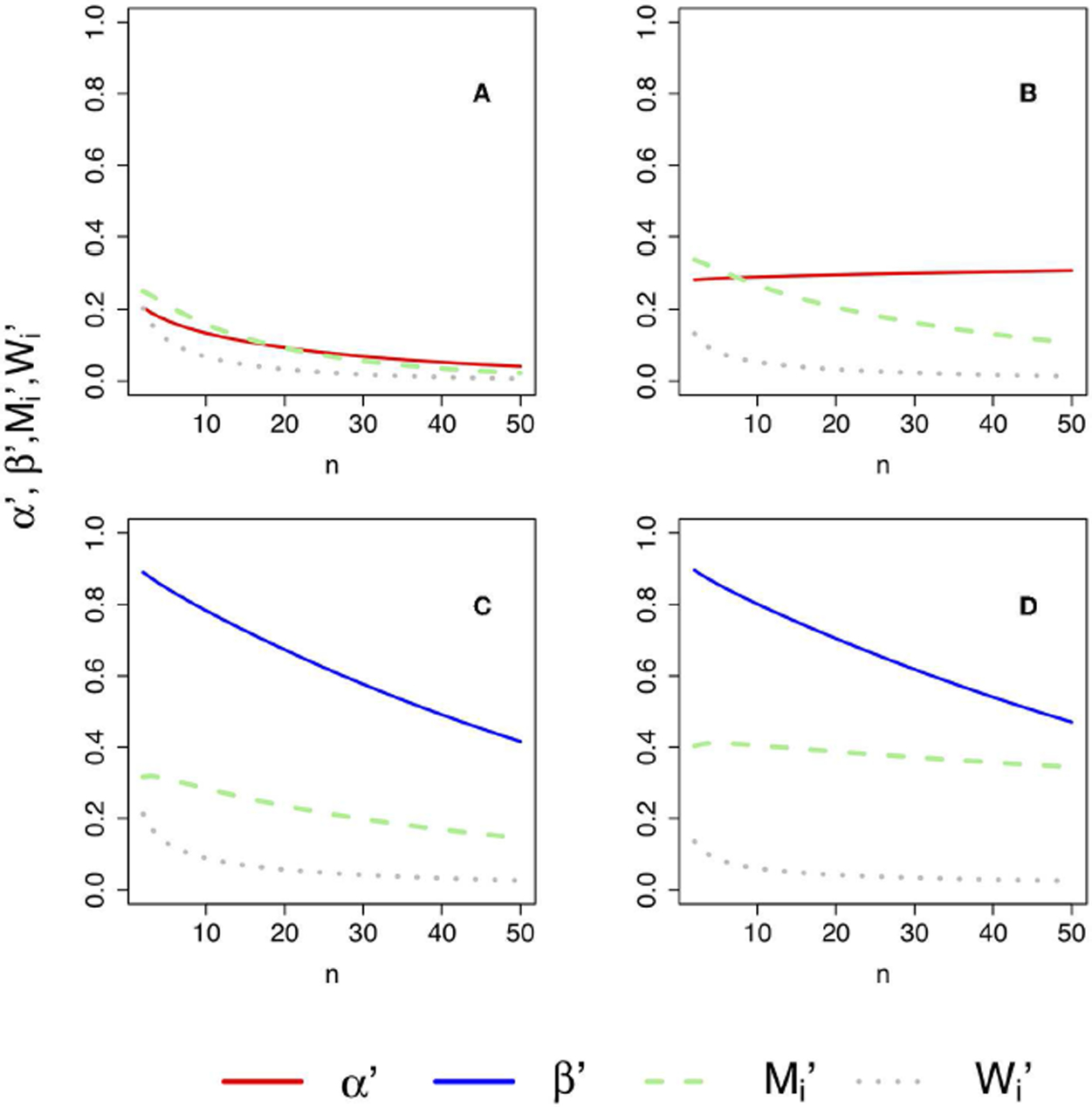
Changes in *α*′ (Equation 53), *β*′ (Equation 59) and $M_{i}^{\prime },W_{i^{\prime }},i=1,2$ (Equations 71, 72) as a function of sample size. The plot in **(A–D)** were computed under each of the geometries plotted in Figures 4A–D. **(A)** *α*′, $M_{1}^{\prime }$, and $W_{1}^{\prime }$ for the models geometry in Figure 4A, where all models are aligned and model *f*_1_ is closest to the generating process. **(B)** Same as in **(A)** but model *f*_1_ is misaligned. **C** *β*′, $M_{2}^{\prime }$, and $W_{2}^{\prime }$ for model geometry in Figure 4C, where model *f*_2_ is closer to the generating process and all models are aligned. D: *β*′, $M_{2}^{\prime }$, and $W_{2}^{\prime }$ for model geometry in Figure 4D, where model *f*_2_ is closer to the generating process but model *f*_2_ is misaligned.

The formulae for *α*′ (Equation 53), *β*′ (Equation 59), and $M_{i}^{\prime },W_{i}^{\prime },i=1,2$ (Equations 71, 72) allow the investigation of how these error rates change as a function of the sample size *n*. However, given that these formulae also involve Δ*K*, *K*_12_, and *K*_21_, multiple configurations should be explored in model space. Figure 4 illustrates how changing parameters can change KL divergences. For instance, the generating process and the approximating models could be aligned in space (see Figure 4A) or not (Figure 4B). Other configurations are explored in Figures 4C,D. The error rates for each one of these configurations are shown in Figure 5.

Four properties of the error probabilities under misspecification are noteworthy. First, *M*_1_′, *M*_2_′, *W*_1_′, and *W*_2_′ all asymptotically approach zero as *n* becomes large provided Δ*K* ≠ 0 (that is, provided one of the models is measurably better than the other), consistent with their behavior under correct specification. Second, for a given value of |Δ*K*|, that is, for a given difference in the qualities of models H_1_ and H_2_ in representing truth, *M*_1_′ is equal to *M*_2_′, and *W*_1_′ is equal to *W*_2_′. Thus, neither model has special standing. Third, *M*_1_′ and *W*_1_′ asymptotically approach *M*_1_ and *W*_1_ as model *f*_1_ becomes better at representing truth (i.e., as *K* (*g*, *f*_1_) → 0), and likewise *M*_2_′ and *W*_2_′ approach *M*_2_ and *W*_2_ as *f*_2_ becomes better. Fourth, if Δ*K* = 0, that is, if both models are equal in quality, then *M*_1_′ and *M*_2_′ each approach 1/2, and *W*_1_′ and *W*_2_′ each approach zero, as *n* becomes large. The above four properties are intuitive and sensible.

The total error probability under misspecification given by *M*_*i*_′ + *W*_*i*_′ (*i* = 1, 2) is identical for both models and remains a monotone decreasing function of *n*:

(73) $${M_{i}}^{\prime }+{W_{i}}^{\prime }\approx \Phi \left(\frac{\sqrt{n}}{\sigma_{g}}\left[\frac{1}{n}\text{log}(k)-\left|\Delta K\right|\right]\right).$$

The probability of strong evidence for model *f*_*i*_ if *f*_*i*_ is closer to *g* is given by *V*_*i*_′ = 1–*M*_*i*_′−*W*_*i*_′ thus remains a monotone increasing function of *n* with an asymptote of 1. As was the case for correctly specified models, *V*_*i*_′ > *M*_*i*_′. Also, *M*_*i*_′ increases at first as a function of *n*, rising to a maximum value before decreasing asymptotically to zero. The value ${\tilde{n}}_{i}^{\prime }$ at which *M*_*i*_′ is maximized is given by

(74) $${\tilde{n}}_{i}^{\prime }=\frac{\text{log}(k)}{\left|\Delta K\right|},$$

with the corresponding maximum value of ${\tilde{M}}_{i}^{\prime }$ being

(75) $${\tilde{M}}_{i}^{\prime }=\Phi \left(-\frac{2\sqrt{\left|\Delta K|\cdot \text{log}(k\right)}}{\sigma_{g}}\right).$$

The expressions for ${\tilde{n}}_{i}^{\prime }$ and ${\tilde{M}}_{i}^{\prime }$ revert to their counterparts *ñ*_*i*_ and ${\tilde{M}}_{i}$ when one of the models is correctly specified. If both models are of equal quality, that is, Δ*K* = 0, then the probabilities *M*_*i*_′ can be considered as probabilities of evidence favoring (wrongly, as the models are a tossup in quality) one or the other models. When Δ*K* = 0, *M*_*i*_′ as a function of *n* has no local maximum and asymptotically approaches 1/2 as sample size increases. The possibility that *M*_*i*_′ might be as great as 1/2 seems distressing, but this only occurs when the two models become equally good (not necessarily identical) approximations of the generating process.

#### 2.2.4. Illustration of Neyman-Pearson Testing and Evidence Under Misspecification

An extension of the Bernoulli example from Figure 3 serves to sharply contrast the error properties of NP testing and evidence analysis. We construct as before two candidate Bernoulli models with respective success probabilities *p*_1_ and *p*_2_. Suppose however that the data actually arise from a Bernoulli distribution with success probability *p*_*g*_. From Equation (17), the value of Δ*K* becomes

(76) $$\Delta K=p_{g}\text{log}\left(\frac{p_{g}}{p_{2}}\right)+\left(1-p_{g}\right)\text{log}\left(\frac{1-p_{g}}{1-p_{2}}\right)-p_{g}\text{log}\left(\frac{p_{g}}{p_{1}}\right)\;-\left(1-p_{g}\right)\text{log}\left(\frac{1-p_{g}}{1-p_{1}}\right)\;=\text{log}\left(\frac{1-p_{1}}{1-p_{2}}\right)+p_{g}\text{log}\left[\frac{p_{1}\left(1-p_{2}\right)}{\left(1-p_{1}\right)p_{2}}\right]$$

Note that Δ*K* is here a simple linear function of *p*_*g*_. In the Figure 3 example, *p*_1_ = 0.75 and *p*_2_ = 0.50. If we take *p*_*g*_ = 0.65, we have a situation in which model 1 is slightly closer to the true model than model 2. As well, we readily calculate that *K*_12_ = 0.130812 and Δ*K* = 0.02095081, so that *K*_12_ > Δ*K*, a situation in which we expect *α*′ to be an increasing function of *n* (as dictated by Equation 53).

**FIGURE 6 |:**
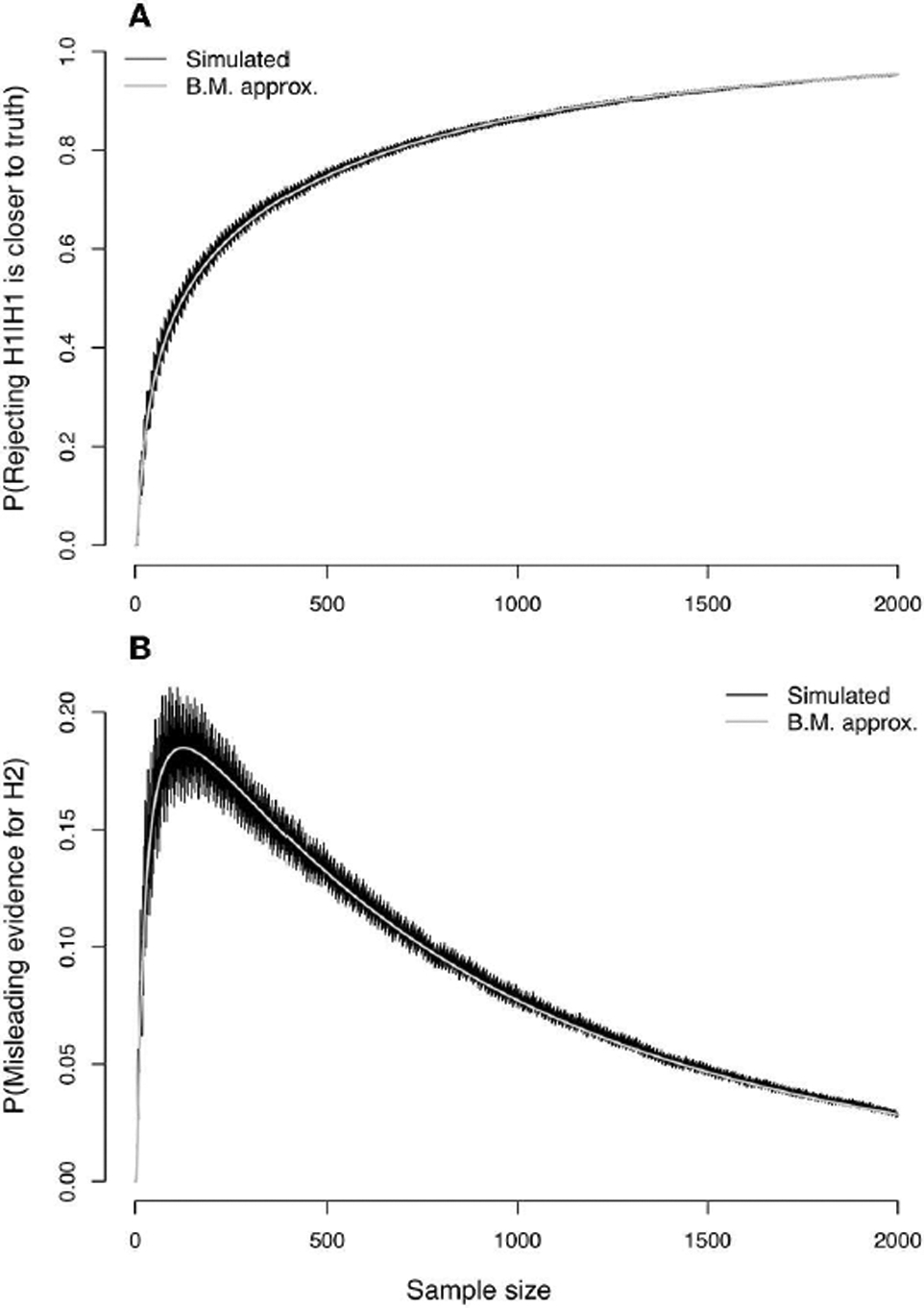
Evidence error probabilities for comparing two Bernoulli(*p*) distributions, with *p*_1_ = 0.75 and *p*_2_ = 0.50, when the true data-generating model is Bernoulli with *p* = 0.65. **(A)** Simulated values (jagged curve) and values approximated under the Central Limit Theorem of the probability (*α*′) of rejecting model H_1_ when it is closer than H_2_ to the true model. **(B)** Simulated values (jagged curve) and approximated values for the probability ($M_{1}^{\prime }$) of misleading evidence for model H_2_ when model H_1_ is closer to the true data-generating process.

The top panel of Figure 6 should give pause to all science. Shown is the probability (*α*′) of wrongly rejecting the null hypothesis of model 1 in favor of the alternative hypothesis of model 2 with Neyman-Pearson testing, under the example scenario of model misspecification in which model 1 is closer to truth. Both simulated values and the CLT approximation (Equation 53) are plotted as a function of sample size. The nominal value of *α* for setting the critical value (*c*) was taken to be 0.05. The curves rapidly approach an asymptote of 1 as sample size increases. With NP testing under model misspecification, picking the wrong model can become a near certainty.

In the bottom panel of Figure 6, the probability of misleading evidence for model 2 ($M_{2}^{\prime }$), that is, of picking the model farther from truth, increases at first but eventually decreases to zero (Figure 6, bottom panel shows simulated values as well as CLT approximation given by Equation 70). Under evidence analysis, the probability of wrongly picking the model farthest from truth converges to 0 as sample size increases.

The example illustrates directly the potential effect of misspecification on the results of the Neyman-Pearson Lemma. The lemma is of course limited in scope, and we should in all fairness note that a classical extension of the lemma to one-sided hypotheses seemingly ameliorates the problem in this particular example. Suppose the two models are expanded: model 1 is the Bernoulli distribution with *p* ≥ 0.75, with model 2 becoming the Bernoulli with *p* < .75. Then, the “Karlin-Rubin Theorem” (Karlin and Rubin, 1956) finds the LR test to be uniformly most powerful size *α* (or less) test of model 1 vs. model 2. Three key ideas enter the proof of the theorem. First, for any particular value *p*_2_ such that *p*_2_ < *p*_1_, the Neyman-Pearson Lemma gives the LR test as most powerful. Second, the cutoff point *c* for the Neyman-Pearson LR test does not depend on the value of *p*_2_. Third, the LR is a monotone function of a sufficient test statistic given by (*x*_1_+*x*_2_+…+*x*_*n*_)/*n*. The upshot is that *α* would remain constant in the expanded scenario, and *β* would decrease toward zero as advertised.

However, the one-sided extension of our Bernoulli example expands the model space to eliminate the model misspecification problem. We regard the hypotheses H_1_: *p* ≥ 0.75 and H_2_: *p* < 0.75 to be a case of two non-overlapping models (Figures 1, 2, bottom) which may or may not be correctly specified. The Karlin-Rubin Theorem would govern if the models are correctly specified. Misspecification in this one-sided context would be exemplified, for instance, by data arising from some other distribution family besides the Bernoulli(*p*), such as an overdispersed family like a beta-Bernoulli (Johnson et al., 2005). Under misspecification, Karlin-Rubin lacks jurisdiction.

#### 2.2.5. Evidence Functions

Lele (2004) took Royall’s (Royall, 1997) approach to using the LR for model comparison and generalized it into the concept of evidence functions. Evidence functions are developed mathematically from a set of desiderata that effective measures of evidence intuitively should satisfy (see Taper and Ponciano, 2016).

The basic insight is that the log-LR emerges as the function to use for model comparison when the discrepancy between models is measured by the KL divergence (Equation 3). The reason is that (1/*n*) log (*L*_1_/*L*_2_) is a natural estimate of Δ*K*, the *difference of divergences of f*_1_ (*x*) *and f*_2_ (*x*) *from truth g* (*x*). However, numerous other measures of divergence or distance between statistical distributions have been proposed (see Lindsay, 2004; Pardo, 2005; Basu et al., 2011), the KL divergence merely being the most well-known. Each measure of divergence or distance would give rise to its own evidence function. Lele (2004) defines an evidence function for a given divergence measure as a data-based estimate of the difference of divergences of two approximating models from the underlying process that generated the data. The motivating idea is to use the data to estimate which of two models is “closer” in some sense to the data generating process. The evidence function concept requires a measure of divergence of a model *f* (*x*) from the true data generating process *g* (*x*) and a statistic, the evidence function, for estimating the difference of divergences from truth of two models *f*_1_ (*x*) and *f*_2_ (*x*). Important among the desiderata for evidence functions (Taper and Ponciano, 2016) is that the probabilities of strong evidence *as defined under misspecification* should asymptotically approach 1 as sample size increases (and so the error probabilities as embodied in *M*_1_′, *M*_2_′, *W*_1_′, and *W*_2_′ would approach zero). It is noteworthy that the prospect of model misspecification is baked into the very definition of an evidence function.

Lele (2004) further proved an optimality property of the LR as evidence function similar to the optimality of the LR in the Neyman-Pearson Lemma. Lele’s Lemma states that, out of all evidence functions, asymptotically, that is for large sample sizes, the probability of strong evidence is maximized by the LR. The result combines the Neyman-Pearson Lemma of hypothesis tests with Fisher’s lower bound for the variance of estimators (see Rice, 2007), extending both. Thus, the information in the data toward quantifying evidence is captured the most by the LR statistic or, equivalently, KL divergence. Other divergence measures, however, have desirable properties, such as robustness against outliers. Modified profile likelihood and conditional likelihood also lead to desirable evidence functions that can account for nuisance parameters, although these modifications to the original LR statistics still are unexplored in terms of their optimality.

## 3. EVIDENCE FUNCTIONS FOR MODELS WITH UNKNOWN PARAMETERS

### 3.1. Information-Theoretic Model Selection Criteria

The latter part of the 20th Century saw some statistical developments that made inroads into the problems of models with unknown parameters (composite models), multiple models, model misspecification and non-nested models, among the more widely adapted of which were the model selection indexes based on information criteria. The work of Akaike (Akaike, 1973, 1974, Figure 7) revealed a novel way of formulating the model selection problem and ignited a new statistics research area. Akaike’s ideas found immediate use in the time series models of econometrics (Judge et al., 1985), were studied and disseminated for statistics in general by Sakamoto et al. (1986) and Bozdogan (1987) and popularized, especially in biology, by Burnham and Anderson (2002).

The information criteria are model selection indexes, the most widely used of which is the AIC (originally, “an information criterion,” Akaike, 1981; now universally “Akaike information criterion”). The AIC is minus two times the maximized log-likelihood for a model, the maximization taken across unknown parameters, with a penalty for the number of unknown parameters added in: ${\text{AIC}}_{i}=-2\text{log}\left({\hat{L}}_{i}\right)+2r_{i}$, where ${\hat{L}}_{i}$ is the maximized likelihood for model H_*i*_, and *r*_*i*_ is the number of unknown parameters in model H_*i*_ that were estimated through the maximization of *L*_*i*_. We are now explicitly considering the prospect of more than two candidate models, although each evidential comparison will be for a pair of models.

Akaike’s fundamental intuition was that it would be desirable to select models with the smallest “distance” to the generating process. The distance measure he adopted is the KL divergence. The log-likelihood is an estimate of this distance (up to a constant that is identical for all candidate models). Unfortunately, when parameters are estimated, the maximized log-likelihood as an estimate of the KL divergence is biased low. The AIC is an approximate bias-corrected estimate of an expected value related to the distance to the generating process. The AIC is an index where goodness of fit as represented by maximized log-likelihood is penalized by the number of parameters estimated. Penalizing likelihood for parameters is a natural idea for attempting to balance goodness of fit with usefulness of a model for statistical prediction (which starts to break down when estimating superfluous parameters). To practitioners, AIC is attractive in that one calculates the index for every model under consideration and selects the model with the lowest AIC value, putting all models on a level playing field so to speak.

Akaike’s inferential concept underlying the AIC represented a breakthrough in statistical thinking. The idea is that in comparing model H_*i*_ with model H_*j*_ using an information criterion, both models are assumed to be misspecified to some degree. The actual data generating mechanism cannot be represented exactly by any statistical model or even family of statistical models. Rather, the modeling process seeks to build approximations useful for the purpose at hand, with the left-out details deemed negligible by scientific argument and empirical testing.

Although AIC is used widely, the exact statistical inference presently embodied by AIC is not widely understood by practitioners. What Akaike showed is that under certain conditions −AIC_*i*_/(2*n*) is (up to an unknown constant) an approximately unbiased estimator of ${\text{E}}_{g}\left\{K\left[g(x),f_{i}\left(x,{\hat{\theta }}_{i}\right)\right]\right\}$, where *θ*_*i*_ is a vector of unknown parameters and ${\hat{\theta }}_{i}$ is its ML estimate, the parameter penalty in AIC being the approximate bias correction. The expectation has two variability components, (1) the distribution of $f_{i}\left(X,{\hat{\theta }}_{i}\right)$ given the ML estimate value, and (2) the distribution of the ML estimate, both expectations with respect to truth *g* (*x*) (In Akaike’s formulation, truth was a model *f*(_■_) with some high-dimensional unknown parameter, while all the candidate models are also in the same form *f*(_■_) except with the parameter vector constrained to a lower-dimensional subset of parameter space. Truth in Akaike’s approach is as unattainable as *g* (*x*)). The double expectation is termed the “mean expected log-likelihood.” The difference AIC_*i*_ − AIC_*j*_ then is a *point estimate* of which model is closer on average to truth, in the sense estimating (−2*n*) times the difference of mean expected log-likelihoods. The approximate bias correction incorporated in AIC is technically correct only if $f_{i}\left(x,{\hat{\theta }}_{i}\right)$ is rather “close” to *g* (*x*); Takeuchi (1976) subsequently provided a mathematically improved (but statistically more difficult to estimate) approximation. “Information theoretic” indexes for model selection have proliferated since, with different indexes refined to perform well for different sub-purposes (Claeskens and Hjort, 2008).

In practice, the AIC-type inference represents a relative comparison of two models, not necessarily nested or even in the same model family, requiring only the same data and the same response variable to implement. The inference is post-data, in that there are (as yet) no appeals to hypothetical repeated sampling and error rates. All candidate models, or rather, all pairs of models, can be inspected simultaneously simply by obtaining the AIC value for each model. But, as is the case with all point estimates, without some knowledge of sampling variability and error rates we lack assurance that the comparisons are informative.

### 3.2. Differences of Model Selection Indexes as Evidence Functions

We propose that information-based model selection indexes can be considered as generalizations of LR evidence to models with unknown parameters, for model families obeying the usual regularity conditions for ML estimation. The evidence function concept clarifies and makes accessible the nature of the statistical inference involved in model selection. Like LR evidence, one would use information indexes to select from a pair of models, say *f*_1_ (*x*, *θ*_1_) and *f*_2_ (*x*, *θ*_2_), where *θ*_1_ and *θ*_2_ are vectors of unknown parameters. Like LR evidence, the selection is a post-data inference. Like LR evidence, the prospect of model misspecification is an important component of the inference. And critically, like LR evidence, the error probabilities *W*_*i*_ and *M*_*i*_ (*i* = 1, 2) can be defined for the information indexes and can in principal be calculated (or simulated) as discussed below. Additionally, as discussed below, many of the existing information indexes retain the desirable error properties of evidence functions. Oddly, the AIC itself does not.

### 3.3. Nested Models, Correctly Specified

As noted earlier, the generalized LR framework of two nested models under correct model specification is a workhorse of scientific practice and a prominent part of applied statistics texts. It is worthwhile then in studying evidence functions to start with the generalized LR framework, in that the model selection indexes are intended in part to replace the hierarchical sequences of generalized LR hypothesis testing (stepwise regression, multiple comparisons, etc.) for finding the best submodel within a large model family.

The model relationships diagrammed in the top portion of Figure 1 depict the two cases. In case 1 (top left), a parameter vector in model *f*_1_ identifies the true model giving rise to the data. Technically the parameter vector is contained in model *f*_2_ as well, but the scientific interest focuses on whether the additional parameters in the unconstrained parameter space of *f*_2_ can be usefully ignored. Case 2 (top right) portrays the situation in which the true parameter vector is in the unconstrained parameter space of model *f*_2_; model *f*_1_ is too simple to be useful.

**FIGURE 8 |:**
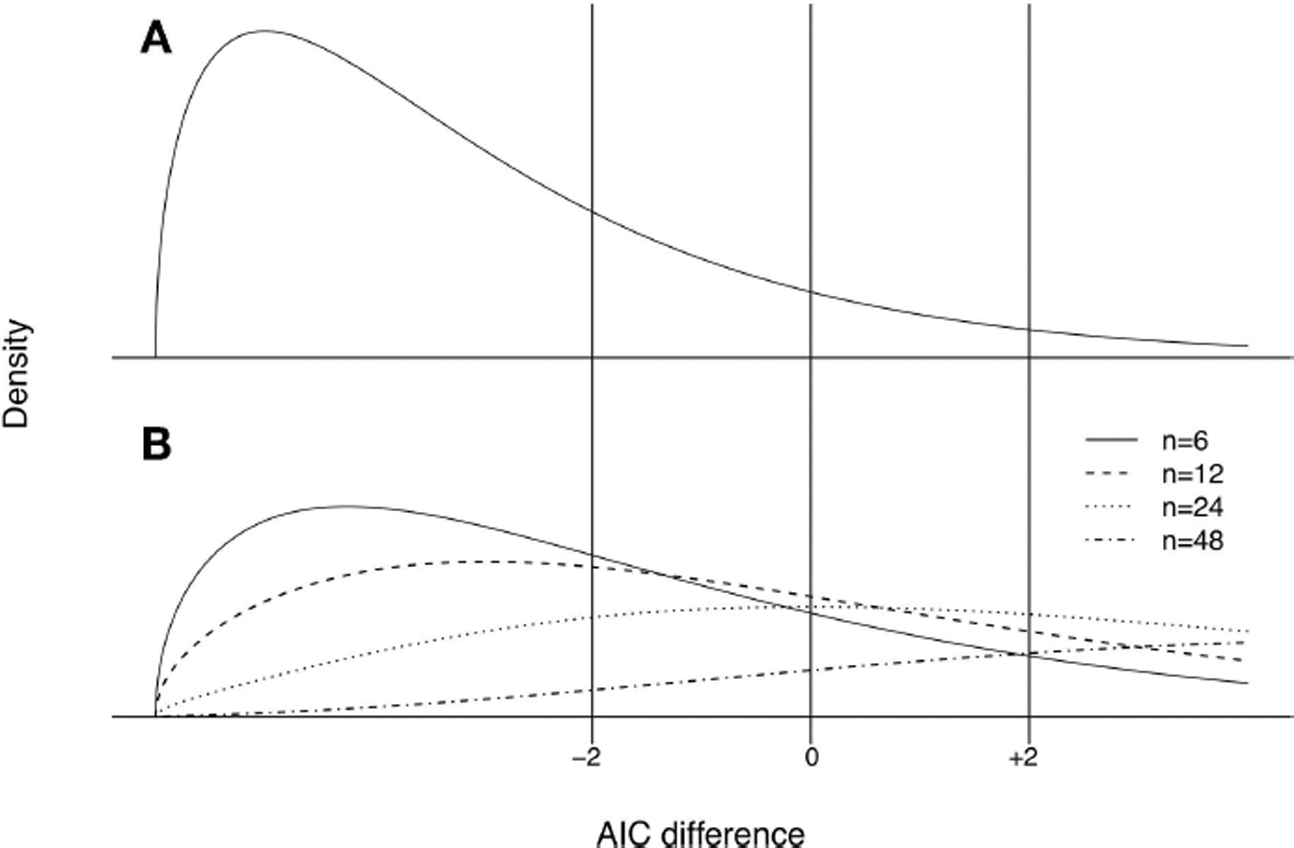
**(A)** Location-shifted chisquare distribution of the difference of AIC values, when data arise from model 1 nested within model 2. In this plot, the degrees of freedom for this distribution are equal to *ν* = 3, and the shift to the left of 0 is equal 2*ν* = 6 (see Equation 77 and text below it). This chisquare distribution is invariant to sample size. As a result, the areas under this distribution in the intervals (−2, +2) and (+2, ∞) corresponding to *W*_1_ and *M*_1_, respectively, are invariant to sample size. **(B)** Non-central chisquare distribution of the difference of AIC values, when data arise from model 2 (but not model 1), plotted for different sample sizes. This distribution is also location-shifted but its non-centrality parameter *λ*, which determines both its mean and variance, is proportional to sample size. In this illustration, *λ* = *n*(1/4). As a result, the areas under the intervals (−2*ν*, −2) and (−2, +2) corresponding to the error probabilities *M*_2_ and *W*_2_ decrease as the sample size increases.

Suppose we decide to use ΔAIC_12_ = AIC_1_ − AIC_2_ as an evidence function. For convenience, we have defined this AIC-based evidence function to vary in the same direction as *G*^2^ (Equation 31) in NP hypothesis testing, so that large values of ΔAIC correspond to large evidence for *f*_2_ (opposite to the direction for the ordinary LR-evidence function given by Equation 33). For instance, the early rule of thumb in the AIC literature was to favor model *f*_1_ when ΔAIC_12_ ≤ −2 and to favor model *f*_2_ when ΔAIC_12_ ≥ 2. Note that

(77) $$\Delta {\text{AIC}}_{12}=G^{2}-2v,$$

where *ν* = *r*_2_ − *r*_1_, the difference of the numbers of unknown parameters in the two models. The behavior of our candidate evidence function ΔAIC_12_ can be studied using the Wilks/Wald results for the asymptotic distribution of *G*^2^. Under case 1, ΔAIC_12_ has (approximately) a chisquare(*ν*) distribution that has been location-shifted to begin at −2*ν* instead of at 0 (top of Figure 8). Under case 2, ΔAIC_12_ has (approximately) a non-central chisquare(*ν*, *λ*) distribution with the same −2*ν* location shift (bottom of Figure 8). The areas under the shifted chisquare pdf in the intervals (−2, + 2) and (+2, ∞) are respectively the generalized error probabilities *W*_1_ and *M*_1_ (Figure 8, top). Likewise, the areas under the shifted non-central pdf in the intervals (−2*ν*,−2) and (−2,+2) are respectively the generalized error probabilities *M*_2_ and *W*_2_ (Figure 8, bottom).

As sample size increases, the error probabilities *W*_1_ and *M*_1_ for the AIC-based evidence function do not go to zero but rather remain positive (Figure 8, top). The value of *n* appears nowhere in the location-shifted chisquare pdf for ΔAIC_12_, and so the error probabilities *W*_1_ and *M*_1_ remain static. Thus, for the AIC, the probabilities of weak and misleading evidence given model *f*_1_ generates the data both behave like the Type 1 error probability *α* in Neyman-Pearson testing. The simulation results of Aho et al. (2014) showing a Type-1-like behavior of the AIC with increasing sample size for particular statistical models are thereby explained (see also Taper and Ponciano, 2016).

As sample size increases, the error probabilities *W*_2_ and *M*_2_ for the AIC-based evidence function do go to zero (Figure 8, bottom). The non-centrality parameter *λ* in the location-shifted non-central chisquare pdf for ΔAIC_12_ is proportional to the value of *n*, and the mean (*ν* + *λ*) of the non-central distribution increases faster than the standard deviation ([2 (*ν* + 2*λ*)]^1/2^), driving the error probabilities *W*_2_ and *M*_2_ to zero. Thus, for the AIC, the probabilities of weak and misleading evidence given model *f*_2_ generates the data both behave like the Type 2 error probability *β* in Neyman-Pearson testing.

Thus, within the generalized likelihood ratio framework, the AIC appears to bring no particular improvement in the sense of evidence to ordinary Neyman-Pearson testing using *G*^2^. Indeed, at least in the Neyman-Pearson approach, the value of *α* is fixed by the investigator and is therefore *known* if the models are correctly specified. The error probabilities attending the use of AIC however are unknown, as they generally are in evidence functions, although they in principle can be estimated with simulation. AIC-based model selection does not have the error properties of an evidence function within the classical milieu of nested statistical models.

Other information-theoretic indexes used for model selection, however, do have performance characteristics of evidence functions. Consider the Schwarz information criterion (SIC; also known as Bayesian information criterion or BIC) given by

$${\text{SIC}}_{i}=-2\text{log}\left({\hat{L}}_{i}\right)+r_{i}\text{log}(n).$$

The index originally had a Bayesian-based derivation (Schwarz, 1978), but its frequentist error properties when employed as an evidence function become apparent with the methods used above for the AIC. As with the AIC, the evidence function version of the SIC would use the difference of SIC values:

$$\Delta \text{SI}{\text{C}}_{12}=\text{SI}{\text{C}}_{1}-\text{SI}{\text{C}}_{2}=G^{2}-v\text{log}(n).$$

**FIGURE 9 |:**
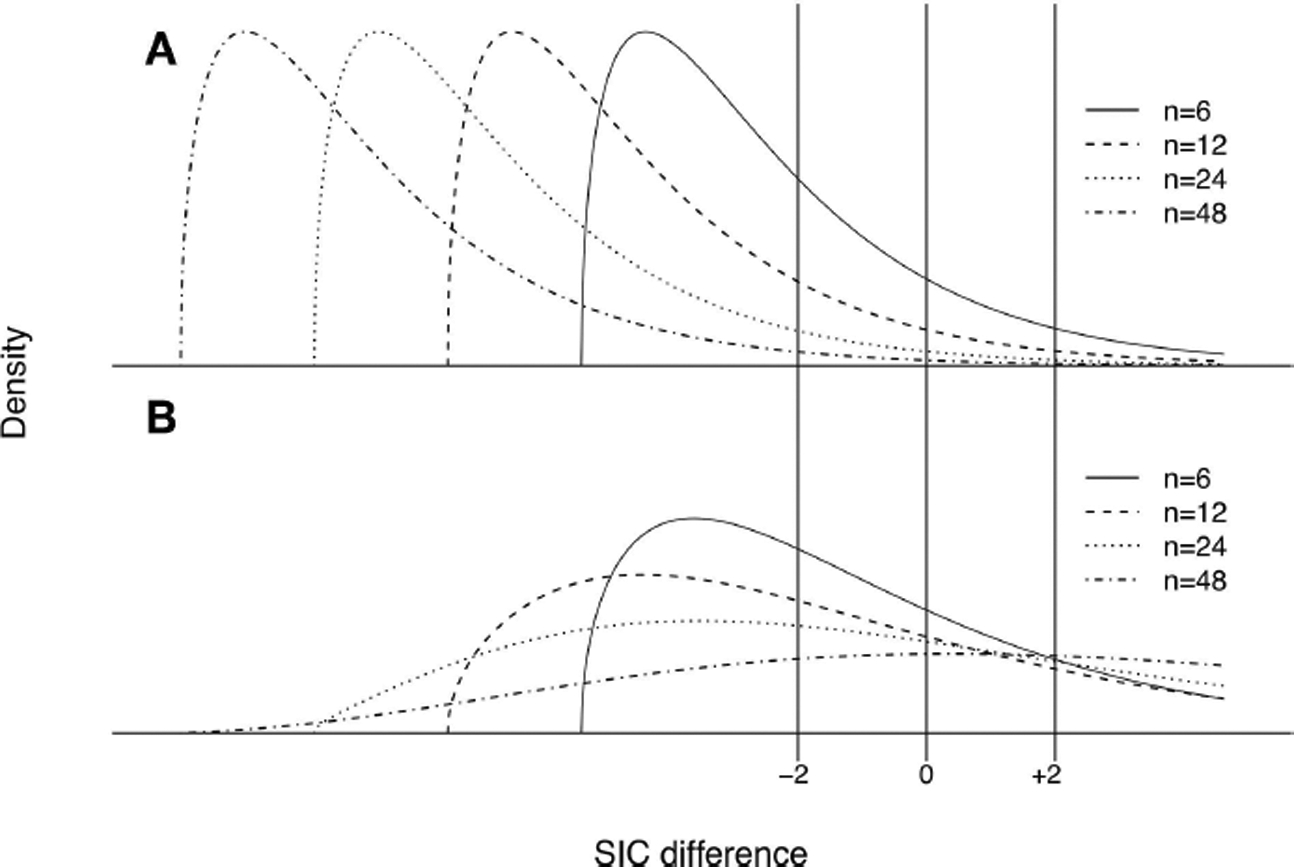
**(A)** Chisquare distribution of the difference of SIC values, when data arise from model 1 nested within model 2. The chisquare distribution is shifted left as sample size increases. **(B)** Non-central chisquare distribution of the difference of SIC values, when data arise from model 2 (but not model 1), plotted for increasing sample sizes.

As with the AIC also, the asymptotic distributions of the SIC evidence function under model *f*_1_ and model *f*_2_ are respectively, location-shifted chisquare and non-central chisquare distributions. For the SIC though, the location of the lower bound of the two distributions at −*ν*log (*n*) decreases as sample size increases (Figure 9, top). If the data arise from model *f*_1_, the chisquare distribution is pulled to the left, and the areas under the pdf corresponding to and eventually decrease asymptotically to zero. If the data arise from model *f*_2_, although the non-central chisquare distribution is also pulled to the left at a rate proportional to log (*n*), the mean is pulled to the right at a rate proportional to *n*, and the coefficient of variation around the mean goes to zero at a rate $1/\sqrt{n}$. The areas under the pdf corresponding to *W*_2_ and *M*_2_ eventually decrease asymptotically to zero (Figure 9, bottom). Thus, unlike the AIC, for nested, correctly specified models the SIC possesses a key quality of an evidence function: all the probabilities of weak and misleading evidence eventually decrease asymptotically to zero.

### 3.4. Misspecified Models

To be fair, AIC as well as evidence functions were forged in the fiery world of misspecified models. Does the AIC difference gain the properties of an evidence function when neither *f*_1_ nor *f*_2_ give rise to the data?

If the models are nested or overlapping, the answer is no. To understand this, we must appeal to modern statistical advances in the theory of maximum likelihood estimation and generalized likelihood ratio testing when models are misspecified. The relevant and general theory can be found in White (1982), Nishii (1988), Vuong (1989), and references therein.

Suppose a model with pdf *f* (*x*, *θ*) is fitted using ML estimation to observations that came from a distribution with pdf *g* (*x*). Under a variety of regularity conditions on the pdfs, the ML estimate has an asymptotic multivariate normal distribution centered on a value θ*, where θ* is the value of *θ* that minimizes *K* (*g* (*x*), *f* (*x*, *θ*)) (White, 1982). The multivariate normal distribution furthermore concentrates around θ* as *n* becomes large, reflecting the fact that the ML estimate under misspecification is a statistically consistent estimate of (converges in probability to) θ*.

Now, any two models *f*_1_ (*x*, *θ*_1_) and *f*_2_ (*x*, *θ*_2_) being compared will be in one of nested, overlapping, or non-overlapping configurations (see Figure 2). Under misspecification in each case, the truth *g* (*x*) is out there, somewhere. We now ask of an evidence function: “Which model contains a parameter set that brings it closer to truth? Is *K* (*g* (*x*), *f*_1_ (*x*, *θ*_1_*)) smaller than *K* (*g* (*x*), *f*_2_ (*x*, *θ*_2_*)) or vice versa?”

The question needs modification in the nested and overlapping cases. If *f*_1_ is nested within *f*_2_, *K* (*g* (*x*), *f*_1_ (*x*, *θ*_1_*)) cannot be smaller than *K* (*g* (*x*), *f*_2_ (*x*, *θ*_2_*)). The modified question becomes “Is *f*_1_ (*x*, *θ*_1_*) as close to truth as *f*_2_ (*x*, *θ*_2_*)?” The question in the nested case is a natural extension of the question asked under correct specification. In the nested case, *K* (*g* (*x*), *f*_1_ (*x*, *θ*_1_*)) being the same as *K* (*g* (*x*), *f*_2_ (*x*, *θ*_2_*)) signifies that *f*_1_ (*x*, *θ*_1_*) and *f*_2_ (*x*, *θ*_2_*) are the same model. If *f*_1_ overlaps *f*_2_, the model closest to truth could be the overlapping region, *K* (*g* (*x*), *f*_1_ (*x*, *θ*_1_*)) would be the same as *K* (*g* (*x*), *f*_2_ (*x*, *θ*_2_*)), and *f*_1_ (*x*, *θ*_1_*) and *f*_2_ (*x*, *θ*_2_*) would be the same model. However, in the overlapping case, *K* (*g* (*x*), *f*_1_ (*x*, *θ*_1_*)) being the same as *K* (*g* (*x*), *f*_2_ (*x*, *θ*_2_*)) does not necessarily signify that *f*_1_ (*x*, *θ*_1_*) and *f*_2_ (*x*, *θ*_2_*) are the same model. The question in the overlapping case becomes “Is the best model in the overlapping region?”

**FIGURE 10 |:**
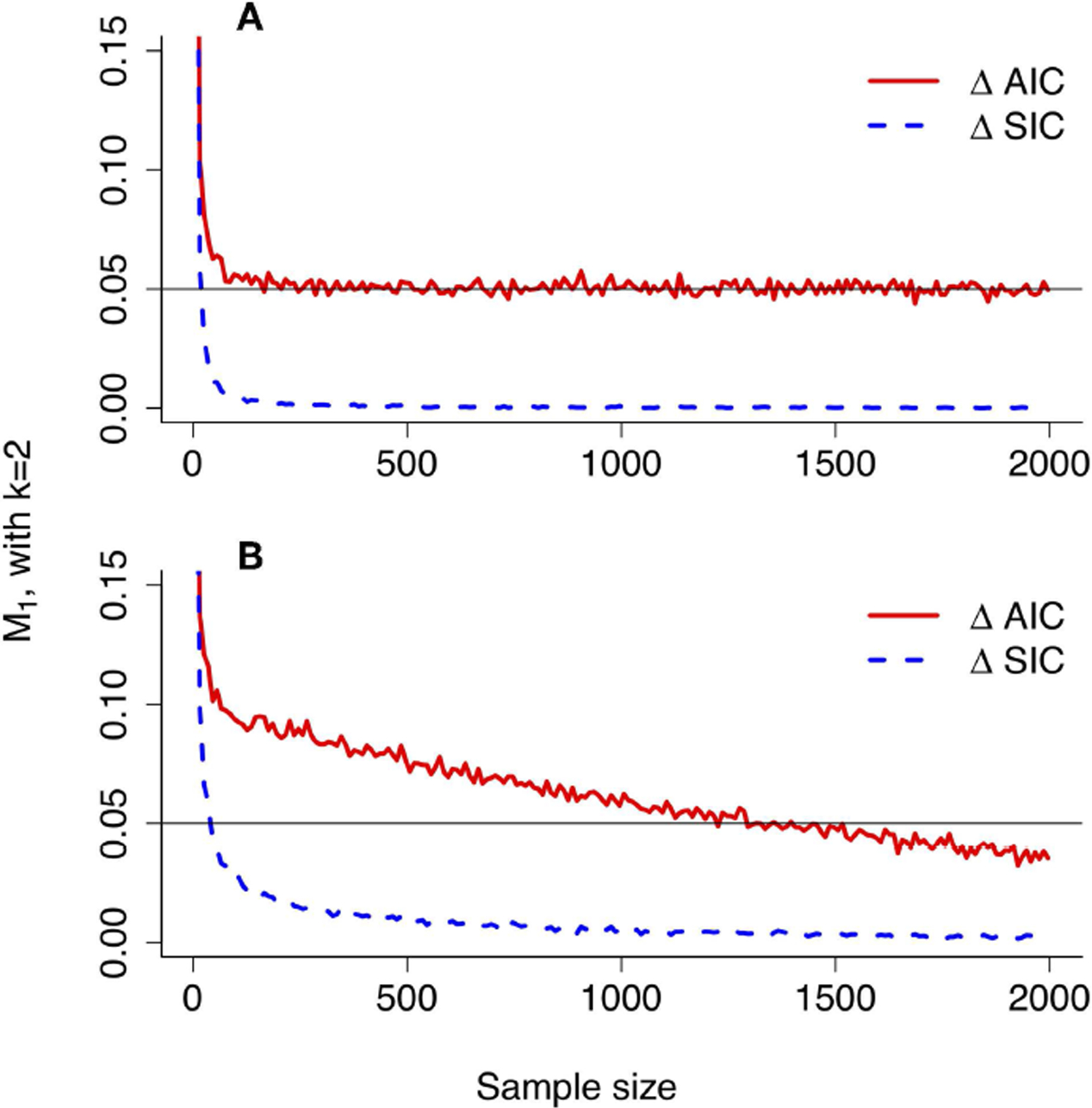
Simulation of Vuong (1989) results for misspecified models. **(A)** When *f*_1_ (*x*, *θ*_1_*) and *f*_2_ (*x*, *θ*_2_*) are the same model (either *f*_1_ is nested within *f*_2_, or *f*_1_ overlaps *f*_2_, and the best model is in the nested or overlapping region), then the asymptotic distribution of *G*^2^ is a “weighted sum of chisquares” that does not depend on *n*. The error probabilities *M*_1_ and *W*_1_ do not decrease to 0 for Δ*AIC*_12_ but do decrease for Δ*SIC*_12_. **(B)** When the models are nested, overlapping, or non-overlapping, but a non-overlapping part of *f*_1_ or *f*_2_ is closer to truth, then *G*^2^ has an asymptotic normal distribution with mean and variance that depend on the sample size, and the error probabilities *M*_1_ and *W*_1_ decrease to 0 for both Δ*AIC*_12_ and Δ*SIC*_12_. Details of these two settings in **(A,B)** are found in a fully commented R code.

Vuong (1989) derived the asymptotic distributions of *G*^2^ under the nested, overlapping, and non-overlapping cases in the presence of misspecification. His main results relevant here are the following, presented in our notation:

- When *f*_1_ (*x*, *θ*_1_*) and *f*_2_ (*x*, *θ*_2_*) are the same model (either *f*_1_ is nested within *f*_2_, or *f*_1_ overlaps *f*_2_, and the best model is in the nested or overlapping region), then the asymptotic distribution of *G*^2^ is a “weighted sum of chisquares” in the form $2a_{j}Z_{j}^{2}$, in which the *Z*_*j*_ are independent, standard normal random variables (each $Z_{j}^{2}$ being chisquare with 1 df) and the *a*_*j*_ values are eigenvalues of a square matrix (*r*_1_ × *r*_2_ rows) of expected values of various derivatives of the two log-pdfs with respect to the parameters (generalization of the Fisher information matrix). The point is, the asymptotic distribution of *G*^2^ does not depend on *n*. ΔAIC_12_ and ΔSIC_12_, along with evidence functions formed from other information indexes, then have location-shifted versions of the weighted sum of chisquares distribution. The error probabilities *M*_1_′ and *W*_1_′ defined for AIC become static and do not decrease to zero as *n* becomes large. The error probabilities *M*_1_′ and *W*_1_′ defined for SIC do decrease to zero, because the location quantity decreases as becomes large, pulling the weighted sum of chisquares pdf to the left (similar to the chisquare distribution in Figure 9). This scenario is simulated and then plotted in Figure 10A.
- Suppose the models are nested, overlapping, or non-overlapping, but a non-overlapping part of *f*_1_ or *f*_2_ is closer to truth, that is, when *f*_1_ (*x*, *θ*_1_*) and *f*_2_ (*x*, *θ*_2_*) are not the same model as in Figure 2. Then *G*^2^ has an asymptotic normal distribution with mean 2*n*ΔK* and variance 4*nσ*_*g*_^2^*, where
  (78) $$\Delta K^{*}=K\left(g(x),f_{2}\left(x,{\theta_{2}}^{*}\right)\right)-K\left(g(x),f_{1}\left(x,{\theta_{1}}^{*}\right)\right),$$
  and
  (79) $${\sigma_{g}}^{2}*=V_{g}\left\{\text{log}\left[\frac{f_{1}\left(X,{\theta_{1}}^{*}\right)}{f_{2}\left(X,{\theta_{2}}^{*}\right)}\right]\right\}.$$
  The result parallels the CLT results (Equations 20–22) for completely specified models, with the added condition that each candidate model is evaluated at its “best” set of parameters. In this situation, the mean of *G*^2^ increases or decreases in proportion to *n*, while the standard deviation increases only in proportion to $\sqrt{n}$. All of the error probabilities, *M*_1_′, *M*_2_′, *W*_1_′ and *W*_2_′ defined for ΔAIC_12_ as well as for ΔSIC_12_ do decrease to zero as *n* becomes large. This scenario is simulated and plotted in Figure 10B.

We must point out that a generalized Neyman-Pearson test (via simulation/bootstrap) of two non-overlapping models with misspecification can suffer the same fate as the completely specified models in the Neyman-Pearson Lemma. The large sample distribution of *G*^2^, assuming model 1 generates the data, would have a mean involving *K*_12_ (evaluated at true parameter value in model 1 and best parameter value in model 2); the cutoff point *c* and other test characteristics would be obtained from this distribution. Under misspecification, the true asymptotic distribution of *G*^2^ has a mean involving ΔK* (Equation 78). As was the case for the two models in the Neyman-Pearson Lemma (Figure 6), discrepancy between *K*_12_ and ΔK* can cause the generalized Neyman-Pearson test to pick the wrong model with Type 1 error probability approaching 1. The Karlin-Rubin Theorem and the forceful language of uniformly most powerful tests does not rescue Neyman-Pearson testing from derailment when inadequate models are deployed.

Error probabilities going to zero can alternatively be derived as a consequence of the (weak or strong) “consistency” of the model selection index. Consistency here means that the index asymptotically picks the model closest to truth as sample size becomes large. Nishii (1988) studied information indexes in the form $-2\text{log}\left({\hat{L}}_{i}\right)-c_{n}r_{i}$, where the parameter penalty coefficient *c*_*n*_ is a possible function of *n*. The parameter penalty determines whether an information-theoretic index behaves like an evidence function. If *c*_*n*_ grows at a rate < *n* but > log log (*n*) then an information-theoretic index will asymptotically pick the model closest to truth Nishii (1988). The difference of such indexes will therefore behave as an evidence function, as the probabilities of picking any of the contending models go to zero. If, however, the penalty term is constant or asymptotically constant, and the model closest to truth is in a parameter region common to two or more models, then the probabilities of weak and misleading evidence are or become constant. The problematic error properties of Neyman-Pearson testing from the standpoint of evidence are thereby preserved in such model selection indexes. For instance, the AIC-corrected index is (Hurvich and Tsai, 1989).

$${\text{AICc}}_{i}={\text{AIC}}_{i}+2r_{i}\left(r_{i}+1\right)/\left(n-r_{i}-1,\right)$$

in which the correction term is designed to improve the behavior of the index under small sample sizes. However, the correction term asymptotically approaches zero as *n* becomes large, and so AICc reverts to AIC, with all its asymptotic error properties, for large samples.

Thus, for either correctly specified or misspecified models in which the best model is in a region of model space that does not overlap any other model under consideration, ΔAIC_12_ indeed behaves like an evidence function. However, many model selection problems, such as in multiple regression, involve collections of models in which model pairs can be nested or overlapping as well as non-overlapping. ΔAIC_12_ will behave more like Neyman-Pearson hypothesis testing for models within overlapping regions and therefore will not possess evidence function properties. differences of information indexes that adjust *G*^2^ with a constant or asymptotically constant location shift, such as the TIC and AICc will share the Neyman-Pearson properties of ΔAIC_12_ and cannot be regarded as evidence functions. differences of those information indexes, such as SIC that produce a location shift that decreases to −∞ as *n* increases (provided that rate is within the Nishii (1988) bounds) will have the error properties of evidence functions.

## 4. DISCUSSION

### 4.1. Comparing Approaches to Statistical Inference

We have shown that key inferential characteristics for Fisher significance analysis, Neyman-Pearson hypothesis testing, and evidential comparison differ substantially. Evidence has inferential qualities that match or surpass Fisher significance and Neyman-Pearson tests (see Table 1):

- *Equal status for both models*. In Fisher significance analysis, there is only one model under consideration. Neyman-Pearson testing compares two models but one of them is accorded special status as the null model and endowed with a fixed error rate (*α*). Evidence analysis compares two models without giving either model special status.
- *Evidence for the null*. Neither Fisher significance analysis nor the conventional form of Neyman-Pearson testing provides evidence for the null hypothesis. Extra analyses (equivalence testing, severity) have been proposed to quantify evidence for the null hypothesis, but such approaches reverse model roles and give special status to the alternative hypothesis. In evidence analysis, one statistic called an evidence function quantifies the evidence for one model and against each of the models in the model set.
- *Accommodates multiple models*. Under Fisher significance analysis, the *P*-values for different models are based on different sufficient statistics and are not strictly comparable. One could compare multiple *P*-values using a shared goodness of fit statistic (not necessarily sufficient), such as the Kolmogorov-Smirnoff. However, pure goodness of fit favors overparameterization (overfitting). Neyman-Pearson testing has been jury-rigged in various forms (stepwise regression, multiple comparisons) to sort through multiple models, but the results at best have only had fair statistical properties. With evidence analysis, all pairs of candidate models can be compared, and thereby all candidate models can be ranked.
- *All error rates go to zero*. Neyman-Pearson testing fixes the Type 1 error probability to be constant, thereby structuring the error rate to be constant regardless of sample size. Fisher significance analysis acquires such a constant error rate when the decision to reject a model is based on a threshold for the *P*-value. Under evidence analysis all error rates approach zero asymptotically with increasing sample size.
- *Total error monotonically decreasing*. In evidence analysis, the total error under each model (1 minus the probability of strong evidence under the model) decreases monotonically and asymptotically to zero with increasing sample size. Because of the special status of the null hypothesis in Neyman-Pearson testing, the total error rate is the Type 1 error rate which remains constant. Fisher significance analysis dons the Type 1 error properties of Neyman-Pearson testing if the decision to reject the model is based on a *P*-value threshold.
- *Non-nested models*. Fisher significance analysis deals with one model at a time, so the idea of comparing two non-nested models is not applicable. The standard extensions (such as generalized likelihood ratio) of the original Neyman-Pearson framework to models with unknown parameters assume that one of the models is nested within the other. Evidence analysis compares two models regardless of their nested or non-nested configuration.
- *Evidence and errors rates distinguished*. The interpretation of a *P*-value has long been a source of confusion among scientists. Because the *P*-value is calculated under the properties of just one model, it is not satisfactory as a measure of evidence for one model over another (Royall, 1986, 1997). Evidence analysis regards error rates and evidence as separate concepts. The evidence approach clarifies *P*-values as error rates defined post-data (see section 2.1.7).
- *Robustness to model misspecification*. Evidence functions are defined in terms of the misspecification of two candidate models. Evidence functions are statistical estimates of which of two models is closer to the true data-generating process. The error rates of evidence analysis, defined robustly as the probabilities of wrong conclusions about which model is closer, go to zero as sample size increases, even under model misspecification. Under model misspecification, Neyman-Pearson testing can fail spectacularly: the Type 1 error rate, defined as the probability of wrongly picking the alternative hypothesis model when the null hypothesis model is just as close to truth, can approach 1 asymptotically as sample size increases. Fisher significance analysis, being in essence a test of whether a given model is misspecified, can be considered to be defined under a presumption of misspecification.
- *Promotes exploration of new models*. Perhaps the most important property of evidence analysis in scientific endeavors is that it explicitly encourages discovery of new models that are closer to truth than models already analyzed. An evidence analysis leaves “room at the top,” or the possibility that a new approach could yield a much better model for the data. In the scientific world, the daily *t*-tests and regressions under Neyman-Pearson testing produces an inertia, a perfunctory routine in statistical analysis often characterized by working scientists as “cookbook” in nature. Barnard’s (1949) observation had Bayesian statistics as its target, but his excruciating words apply to any kind of modeling: “To speak of the probability of a hypothesis implies the possibility of an exhaustive enumeration of all possible hypotheses, which implies a degree of rigidity foreign to the true scientific spirit. We should always admit the possibility that our experimental results may be best accounted for by a hypothesis which never entered our own heads.”

### 4.2. Prediction-Efficient vs. Consistent Criteria

#### 4.2.1. Prediction-Efficiency

AIC and its asymptotic relatives like AICc are built around statistical prediction. The difference of mean expected log-likelihoods is different from what we have defined above as ΔK*. The mean expected log-likelihood has a second, predictive layer of expectation in its definition, the idea being to identify the model that could best predict a new observation from *g* (*x*), taking into account the uncertainty in the estimation of unknown parameters. For this reason these criteria have been termed the efficient, asymptotically efficient, or prediction-efficient criteria (Shibata, 1980; Hurvich and Tsai, 1990).

The tendency for AIC related criteria to over fit is a natural consequence of their design goal of prediction mean square error (MSE) minimization. When parameters are estimated, the increase in prediction MSE due to adding a spurious covariate is generally less than the reduction in prediction MSE caused by including a relevant covariate.

The tendency of stepwise regression to overfit using Neyman-Pearson testing has long been noted (Wilkinson and Dallal, 1981; Hurvich and Tsai, 1990; Harrell, 2001; Rao et al., 2001; Blanchet et al., 2008; Mundry and Nunn, 2008). The fixed Type 1 error rate as a criterion for entry (or exit) of a variable is at the heart of the overfitting problem, and methods for altering the Type 1 error rate based on the number of model parameters have been proposed (e.g., Foster and George, 1994). Such interventions without sample size in the recipe do not produce error rates that universally converge to zero as sample size becomes large.

Model selection with AIC or AICc improves somewhat on the Neyman-Pearson overfitting problem in that the misleading error probabilities both go to zero as sample size increases when two non-overlapping models are being compared. However, overlapping models, in which AIC and AICc are prone to overfit, are typically a substantial subset of the models in contention in multiple regression. The AIC and AICc indexes will tend to include spurious variables too often and thus represent only a partial improvement over stepwise regression.

#### 4.2.2. Identifying Causal Structure

Scientific prediction, however, can be broader than pure statistical prediction. The scientist often desires to predict the outcome of a system manipulation: what will happen if harvest rate is increased, or if habitat extent is halved? Modeling such manipulation might translate as a structural change in a statistical model of the system. The predictive quality of the model then lies more in getting mechanisms in the model as right as possible.

The consistent criteria will asymptotically select the generating process if it is in the model set. If the generating process is not in the model set, the consistent criteria will asymptotically select the model in the set that under best possible parameterization is closest (in the KL sense) to the generating process. The estimation of ΔK* by the difference of SIC values represents a quest for a different kind of prediction that might come from a structural understanding of the major forces influencing the system under study. The tendency of the prediction efficient criteria to include spurious covariates promotes a mis-understanding of the generating mechanism (Taper, 2004).

Certainly, the finite-sample properties of SIC and other consistent indexes require substantial further study, but the property that more data should be able to distinguish among candidate models with fewer errors seems an important property to preserve.

The scientific allure of information-theoretic indexes resided in the idea that all models were evaluated on a level playing field. One would calculate the index for each model and select the model with the best index, a procedure which promised considerably more clarity over hierarchical sequences of Neyman-Pearson tests, such as stepwise regression.

### 4.3. Uncertainty in Evidence

AIC and its descendants were originally built around concepts of statistical point estimation. The statistical inference represented by AIC is that of an approximately unbiased point estimate of the mean expected log-likelihood. The statistical concepts of errors and variability in information indexes have by contrast not often been emphasized. Partly as a result, model selection with information indexes has been somewhat of a black box for investigators, as achieving a good understanding of the inferences represented by model selection analyses is a mathematical challenge (see Taper and Ponciano, 2016).

#### 4.3.1. Evaluating Model Adequacy

We have illustrated that, unlike the error rates in Neyman-Pearson hypothesis testing, all of the error rates of evidence analysis converge to zero as sample size increases. However, the errors we have discussed deal only with the determination which of two models is closer to truth; the error rates do not shed light on whether either model is close enough to truth to be scientifically or managerially valuable. This question is the realm of model adequacy analysis.

Whether the statistical inference is a hypothesis test, equivalence analysis, severity analysis, or evidence analysis, whether for a pair of models or multiple pairs of models, a follow-up evaluation of model adequacy looms ever more important as a crucial step (Mayo and Spanos, 2004; Spanos, 2010). Lindsay (2004) and Markatou and Sofikitou (in review) discuss ideas about the statistical evaluation of model adequacy. Mac Nally et al. (2018) give an impassioned editorial plea for routine model adequacy evaluation in scientific model selection. Ponciano and Taper (2019) show how to directly incorporate model adequacy evaluation into information criterion based model selection.

Considering the likely prevalence of model misspecification in ecological statistics, analysts will need to consider how a candidate model could be misspecified as well as the effects of such misspecification on the intended uses of the model. Practically, the analyst can introduce models formulated in diverse fashions and let the model identification process itself reduce model misspecification. Further experimental or observational tests of model predictions (e.g., Costantino et al., 2005) and their associated error rates are necessary to map the conditions under which a given model is reliable.

The error properties of evidence analysis are more difficult to calculate than classical NP tests because model misspecification is involved. But once calculated, the rates are likely to be more accurate than classical tests that pretend misspecification does not exist.

#### 4.3.2. Approaches to Estimating Post-data Error Rates

Error rates are different pre and post-data. *W*, *M* and *α* are pre-data error rates calculated under a model that is assumed to be true. The *P*-value is a post-data error rate. The pre-data error rates are useful for experimental design, but should be viewed with suspicion as a post-data inference tool because as we have shown these error rates are only accurate if the generating process is the assumed model. Little work has been performed on evidential error rates under the realistic assumption of model misspecification (but see Royall and Tsou, 2003). This area is an important field for future work.

Non-parametric bootstrapping shows great promise for calculating evidential error rates, for data structures that allow bootstrapping. In work in preparation, we (Taper, Lele, Ponciano, and Dennis) show that bootstrapping greatly aids in the interpretation of evidential results. Figures 4, 5 indicate that evidential error rates depend on the structure of the model space. Taper and Ponciano (2016) and Ponciano and Taper (2019) show that given data and a set of models, estimation of the model space structure including the location of the unknown generating process is feasible. This gives a direct measure of model adequacy. Future extensions of this work may allow for the direct estimation of realistic error rates as well.

### 4.4. How Should One Use Evidential Statistics in Practice?

A basic recommendation is to stop using NP tests for inference and be cautious about using the AIC family of information criteria for model selection. These are known as the “efficient” or “MSE minimizing” criteria and include the AIC, the AICc, the TIC, many forms of ICOMP and the EIC. These criteria are recognized by a complexity penalty whose expectation is asymptotically constant. Asymptotically equivalent to the AIC is the use of leave-one-out cross-validation (Stone, 1977); cross-validation will have model selection properties similar to AIC but has the advantage that it can be calculated in the absence of a likelihood function.

There is no reason that the multiple comparisons inference from traditional ANOVAs cannot be made using information criteria (e.g., Kemp et al., 2004; Jerde et al., 2019).

Classical methods will work well for state description and less well for process identification. Unbiased scientific inferences of process are better made using consistent information criteria (see Jerde et al., 2019; Lorah and Womack, 2019 for examples). Analysts have a convenient spectrum of choices for many standard modeling situations in a suite of consistent information criteria: The HQIC (also known as the HQC, Hannan and Quinn, 1979), the HIC (aka BIC* and HBIC, Haughton, 1988), the SIC (aka BIC and SBC, Schwarz, 1978), and the CAIC (Bozdogan, 1987). The analyst can opt for a criterion that matches her goals. The sample size multiplier in the HQIC grows at the minimal rate to generate a consistent form. As a consequence the HQIC will behave very much like the AIC, selecting models with low MSE of prediction by capturing real but small effects at the cost of including spurious covariates. The HIC tends to balance underfitting and overfitting errors. The SIC and CAIC both favor compact models, with all the included components well-supported, and both tend to underfit. The CAIC has the strongest complexity penalty and thus makes the most underfitting errors and the fewest overfitting errors.

Besides being influenced by inferential goals, the choice of evidence function should depend on the modeling framework. Information criteria had their beginnings as a tool for variable selection in linear regression with independent observations. In such situations, as derived by Akaike, the number of parameters is a good first order bias correction to the observed likelihood. But, statistics is a world of special cases. The dizzying diversity of information criteria in the literature produces the desire to optimize the bias correction under different modeling frameworks. For instance, in mixed models, even the meaning of the number of parameters or the number of observations becomes ambiguous due to the dependence structure of mixed models. Information criteria have been developed using estimates of the effective number of parameters (e.g., Vaida and Blanchard, 2005; You et al., 2016). Similarly, information criteria have been constructed using estimates of the effective number of observations (e.g., Jones, 2011; Berger et al., 2014).

If the generating process is in the model set, or in flat model spaces, such as those in linear regression, the ΔAIC is an unbiased estimate of 2*n*Δ*K* regardless of how near or far each of the approximating models is to the generating process (Burnham and Anderson, 2002; Choi and Kiefer, 2011). In curved model spaces (as in Efron, 1975), ΔAIC is not unbiased, and the estimation is only good if both approximating models are close to the generating process. The Takeuchi’s information criterion, the TIC (Takeuchi, 1976; Shibata, 1989), is nearly unbiased even for curved models at great distances from the generating process (Burnham and Anderson, 2002; Choi and Kiefer, 2011). Optimal multiplicative coefficients of bias adjustment for the AIC and TIC have been given (Ogasawara, 2016). Also, Ogasawara showed that when the penalty term in TIC (a random variable, not a constant) is negatively correlated with the main term, the higher-order asymptotic variance of the TIC becomes smaller than that common to the AIC and BIC. Unfortunately, the complexity penalty for the TIC must be estimated from data and cannot be specified a priori, as with the other criteria mentioned. The uncertainty in penalty estimation makes the use of the TIC impractical unless sample size is large. A second problem with the TIC is that like the AIC, it is not consistent, but any efficient information criterion can be made consistent either by multiplying the complexity penalty by a consistent multiplier (Nishii, 1988) or by averaging the penalty with a consistent penalty (Lorah and Womack, 2019). Lorah and Womack (2019) also report on testing a list of various model selection criteria. In a nutshell, model selection criteria made into evidence functions as a whole give reasonable and responsible results, with none of the criteria being universally best. Which evidence function is better depends on the nature of the problem at hand, that is, the characteristics of the model space being investigated. The technical difficulties of criterion selection aside, the most important aspect of applying evidential statistics is approaching problems evidentially.

## 5. CONCLUSION

Evidence is not so much a new statistical method for model selection as it is a new way of thinking about the inference involved with existing model selection methods. The evidential way of thinking has two main components: (1) A post-data trichotomy of outcomes (strong evidence for model *f*_*i*_, weak or inconclusive evidence, strong evidence for model *f*_*j*_). (2) A framework of pre-data error probabilities, which are assured to go to zero as sample size increases. The evidential approach invites exploration of the error probabilities, usually via simulation, to aid in study design, the selection of evidence thresholds, the effects of different types of misspecification, and the interpretation of study results.

We have proposed here a different way of thinking about statistical analyses and model selection, based on the concept of evidence functions. Evidence is an intuitive way to decide between two models that avoids the famously upside-down logic that accompanies Neyman-Pearson testing. Evidential thinking has helped us reveal the shortcomings of Fisher significance analysis and Neyman-Pearson testing. The errors that can arise in evidence analysis are straightforward to explain, and the frequentist properties of such errors as functions of sample size and effect size are easy to understand and highly compelling in a scientific chain of argument. The information indexes, when differenced, represent a collection of potential evidence functions that extend the evidence ideas to models with unknown parameters. The desirable error properties are preserved in the presence of model misspecification, when the model choice is generalized to be an inference about which model is closer to the stochastic process that generated the data. The error properties of AIC and AICc are similar to those of Neyman-Pearson testing when the candidate models are nested or overlapping and so the AIC-type indexes are not satisfactory evidence functions in those common circumstances. The indexes like SIC in which the parameter penalty is an increasing function of sample size retain the frequentist error properties of evidence functions for all model pairs.

Evidence works well for science in part because its explicit conditioning on the model set invites thinking about new models. Evidence has inferential qualities that match or surpass Fisher significance analysis and Neyman-Pearson tests. Evidence represents a compelling scientific warrant for formulating statistical analyses as model selection problems.

## Figures and Tables

**FIGURE 7 |:**
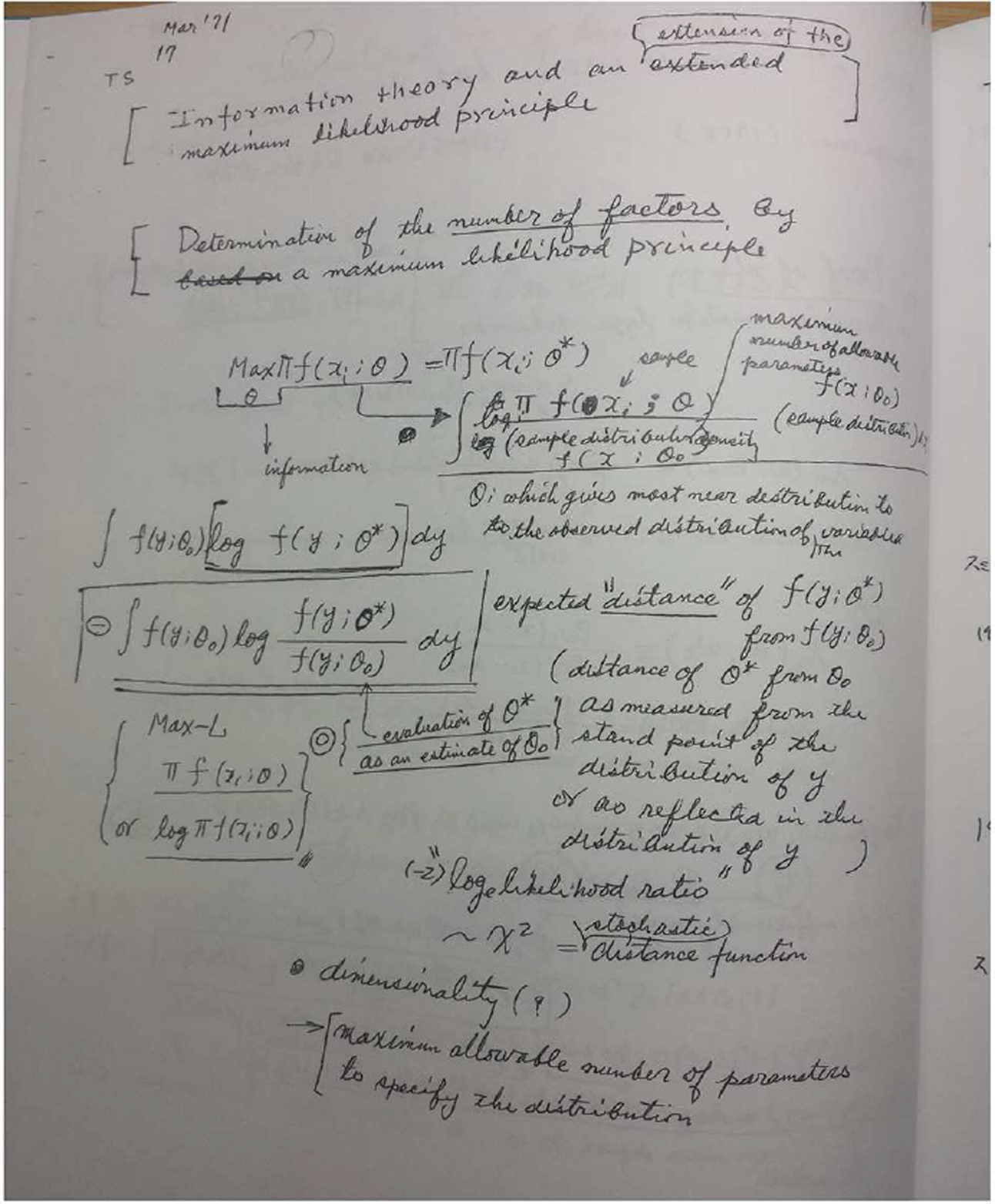
Moment of discovery: page from Professor H. Akaike’s research notebook, written while he was commuting on the train in March 1971. Photocopy kindly provided by the Institute for Statistical Mathematics, Tachikawa, Japan.

**TABLE 1 |:** A comparison of inferential characteristics between Fisherian significance testing (*P*-values *sensu stricto*), Neyman-Pearson hypothesis tests (including *P*-values for likelihood ratios) and evidential statistics.

Inferential characteristic	*P*-value	NP-test	Evidence
Equal status for null and alternatives	NA	No	Yes
Allows evidence for Null	No	No	Yes
Accommodates multiple models	No	Awkward	Yes
All error rates go to zero as sample size increases	No	No	Yes
Total error rate always decreases with increasing sample size	No	No	Yes
Can be used with non-nested models	NA	Not Standard	Yes
Evidence and error rates distinguished	No	No	Yes
Robust to model misspecification	Yes	No	Yes
Promotes exploration of new models	Yes	No	Yes

## BOX 1 | The Central Limit Theorem (CLT)

Suppose that *X*_1_, *X*_2_, … , *X*_*n*_ are independent and identically distributed random variables with common finite mean denoted *μ* = *E*(*X*_*i*_), and finite variance denoted *σ*^2^ = *E*[(*X*_*i*_ − *μ*)^2^]. Let

$$S_{n}=X_{1}+X_{2}+\ldots +X_{n}$$

be the sum of the *X*_*i*_s. Let $\text{P}\left(\frac{S_{n}-n\mu }{\sqrt{n\sigma^{2}}}\leq s\right)=F_{n}\left(s\right)$ be the cumulative distribution function (CDF) for *S*_*n*_ standardized with its mean *nμ* and its variance *nσ*^2^, equivalently written as $\sqrt{n}\left({\bar{X}}_{n}-\mu \right)/\sigma$, where ${\bar{X}}_{n}=\frac{1}{n}S_{n}$. Then as *n* → ∞, *F*_*n*_ (*s*) converges to the cdf of a normal distribution with mean of 0 and variance of 1. We say that $\frac{S_{n}-n\mu }{\sqrt{n\sigma^{2}}}$ converges in distribution to a random variable with a normal (0, 1) distribution, and we write

$$\frac{S_{n}-n\mu }{\sqrt{n\sigma^{2}}}=\frac{\sqrt{n}}{\sigma }\left({\bar{X}}_{n}-\mu \right)\overset{d}{\to }\text{ normal }(0,1).$$

From the CLT one can obtain normally distributed approximations for various quantities of interest:

$$S_{n}\dot{\sim }\text{ normal }\left(n\mu ,n\sigma^{2}\right),$$

$${\bar{X}}_{n}=\frac{1}{n}S_{n}\dot{\sim }\text{ normal }\left(\mu ,\frac{\sigma^{2}}{n}\right).$$

Here, ⩪ means “approximately distributed as.” A general proof of the CLT as presented in advanced mathematical statistics texts typically uses the theory of characteristic functions (Rao, 1973).
